# Hierarchical Compression Reveals Sub-Second to Day-Long Structure in Larval Zebrafish Behavior

**DOI:** 10.1523/ENEURO.0408-19.2020

**Published:** 2020-07-13

**Authors:** Marcus Ghosh, Jason Rihel

**Affiliations:** Department of Cell and Developmental Biology, University College London, London WC1E 6BT, United Kingdom

**Keywords:** behavioral dynamics, sleep, zebrafish

## Abstract

Animal behavior is dynamic, evolving over multiple timescales from milliseconds to days and even across a lifetime. To understand the mechanisms governing these dynamics, it is necessary to capture multi-timescale structure from behavioral data. Here, we develop computational tools and study the behavior of hundreds of larval zebrafish tracked continuously across multiple 24-h day/night cycles. We extracted millions of movements and pauses, termed bouts, and used unsupervised learning to reduce each larva’s behavior to an alternating sequence of active and inactive bout types, termed modules. Through hierarchical compression, we identified recurrent behavioral patterns, termed motifs. Module and motif usage varied across the day/night cycle, revealing structure at sub-second to day-long timescales. We further demonstrate that module and motif analysis can uncover novel pharmacological and genetic mutant phenotypes. Overall, our work reveals the organization of larval zebrafish behavior at multiple timescales and provides tools to identify structure from large-scale behavioral datasets.

## Significance Statement

Behavior is dynamic and not only can change from one second to the next but also can unfold over many hours or even days. Understanding how behavior is organized on these different timescales is a critical task in neuroscience, because the constraints on and patterns of behavior offer important clues about the underlying computations being performed in the brain. The analysis tools we develop in this manuscript and apply from sub-second to day-long larval zebrafish behavior expands our understanding of how behavioral patterns change at multiple timescales. The computational metrics we describe can now be used to understand the behavioral consequences of psychotropic drugs or genetic lesions associated with neurodevelopmental and neuropsychiatric disorders.

## Introduction

To survive, animals must coordinate patterns of action and inaction in response to their environment. These actions and inactions, which together we will define as behavior, result from some function incorporating internal (e.g., transcriptional, hormonal, or neuronal activity) and external (e.g., time of day or temperature) state. Thus, behavioral descriptions provide insight into the underlying mechanisms that control behavior and are a necessary step in understanding these systems (Krakauer et al., 2017).

Animal behavior, however, typically has many degrees of freedom and evolves over multiple timescales from milliseconds (Wiltschko et al., 2015) to days (Proekt et al., 2012; Fulcher and Jones, 2017) and even across an animal’s entire lifespan (Jordan et al., 2013; Stern et al., 2017). As such, quantitatively describing behavior remains both conceptually and technically challenging (Berman, 2018; Brown and de Bivort, 2018). Inspired by early ideas from ethology (Lashley, 1951; Tinbergen, 2010), one approach is to describe behavior in terms of simple modules that are arranged into more complex motifs. Behavioral modules are often defined from postural data as stereotyped movements, such as walking in *Drosophila* (Berman et al., 2014; Vogelstein et al., 2014; Robie et al., 2017) and mice (Wiltschko et al., 2015), while behavioral motifs are defined as sequences of modules, which capture the patterns inherent to animal behavior, such as grooming in *Drosophila* (Berman et al., 2014, 2016).

Zebrafish larvae have emerged as a powerful model organism in neuroscience, owing to their genetic tractability (Howe et al., 2013), translucency (Vanwalleghem et al., 2018), and amenability to pharmacological screening (Rihel and Ghosh, 2015). In terms of behavior, larvae exhibit an alternating sequence of movements and pauses, termed bouts. This structure is particularly suited to modular description as individual bouts can be easily segmented and it is relatively easy to acquire many examples from even a single animal due to the high frequency of their movement (Kim et al., 2017). Leveraging these advantages, recent work used unsupervised learning to uncover a locomotor repertoire of 13 swim types in larval zebrafish, including slow forward swims and faster escape swims (Marques et al., 2018). However, the inactive periods between swim bouts were not considered, despite reflecting behavioral states such as passivity in the face of adversity (Mu et al., 2019) or even sleep (Prober et al., 2006).

To explore an animal’s full behavioral repertoire, from fast movements to sleep, it is necessary to study behavior over long timescales. To date, however, module and motif descriptions of behavior have been developed from videos 15 min (Vogelstein et al., 2014; Wiltschko et al., 2015; Robie et al., 2017) to 2 h (Marques et al., 2018) in length. Consequently, most identified behavioral structure has been on the order of milliseconds, and the existence of longer-timescale structure, on the order of minutes to hours has remained largely unexplored. The development of methods to extract multi-timescale structure from long-timescale recordings would open avenues to explore questions about how behavior varies across the day/night cycle and develops across an animal’s lifespan. Furthermore, as pharmacologically or genetically induced behavioral phenotypes can differ at different times of the day/night cycle in zebrafish larvae (Rihel et al., 2010a; Hoffman et al., 2016), a long-timescale approach would provide valuable phenotyping information.

Currently, the limiting factor in scaling these methods is the volume of data, owing to the high framerates and dimensionality required to estimate animal posture. Here, we present an alternative approach in which we trade dimensionality for scale by building a module and motif description of larval zebrafish behavior from a one-dimensional behavioral parameter recorded over time. Specifically, we used a high-throughput behavioral set-up (Rihel et al., 2010b) to continuously monitor the activity of hundreds of zebrafish larvae across multiple days and nights. To identify multi-timescale behavioral structure, we developed a three-step computational approach. First, we used unsupervised learning to identify a set of 10 behavioral modules that describe both active and inactive bout structure. Second, we applied a compression algorithm (Nevill-Manning and Witten, 2000) to our module data to compile a library of almost 50,000 motifs, revealing behavioral patterns organized across sub-second to minute timescales. Finally, we used a supervised learning algorithm (Peng et al., 2005) to identify motifs from the library used at particular times of the day/night cycle. To test the ability of our approach to detect biologically relevant phenotypes, we also studied the behavior of larvae exposed to the seizure-inducing drug, pentylenetetrazol (PTZ; Baraban et al., 2005), the sedating drug, melatonin (Zhdanova et al., 2001), and mutants of the hypocretin receptor (*hcrtr*; Yokogawa et al., 2007), the loss of which is associated with narcolepsy in humans (Lin et al., 1999) and altered bout structure in zebrafish (Yokogawa et al., 2007; Elbaz et al., 2012). We found that our computational approach could readily detect both compound dose and mutant specific differences in module and motif usage, demonstrating the biological relevance of our behavioral description.

Ultimately, our work reveals the organization of larval zebrafish behavior at sub-second to day-long timescales and provides new computational tools to identify structure from large-scale behavioral datasets.

## Materials and Methods

### Animal husbandry

Adult zebrafish were reared by University College London (UCL) Fish Facility on a 14/10 h light/dark cycle (lights on: 9 A.M. to 11 P.M.). To obtain embryos, pairs of adult males and females were isolated overnight with a divider that was removed at 9 A.M. the following morning. After a few hours, fertile embryos were collected and sorted under a bright-field microscope into groups of 50 embryos per 10-cm Petri dish filled with fresh fish water (0.3 g/l Instant Ocean). Plates were kept in an incubator at 28.5°C on a 14/10 h light/dark cycle. Using a Pasteur pipet under a bright-field microscope, debris was removed from the plates and the fish water replaced each day. All work was in accordance with the United Kingdom Animal Experimental Procedures Act (1986) under Home Office Project License 70/7612 awarded to J.R.

### Behavioral setup

For all behavioral experiments a Pasteur pipet was used to transfer single zebrafish larvae [aged 4–5 d post fertilization (dpf)] into the individual wells of a clear 96-square well plate (7701-1651; Whatman); then each well was filled with 650 μl of fish water. For experiments longer than 24 h, larvae were plated at 4 dpf and tracking was started the same day. For the duration of these experiments, evaporated fish water was replaced each morning between 9 and 9:30 A.M. For the wild-type experiments, each plate was covered with a plastic lid (4311971; Applied Biosystems) to prevent evaporation and to negate the need to replenish the fish water. For the 24-h small molecule experiments (melatonin and PTZ), larvae were plated at 5dpf and the plates were left overnight in a 28.5°C 14 h/10 h light/dark incubator. The following morning each plate was transferred to a behavior setup where larvae were dosed, between 9 and 10 A.M., immediately after which behavioral recordings were started and run for 24 h. Following two control rows, each row was dosed with a successively higher concentration of test compound. Larvae were not fed either before or during experiments.

To record each animal’s behavior, each plate was placed into a Zebrabox (ViewPoint Life Sciences) running quantization mode with the following settings: detection sensitivity, 15; burst, 50; and freezing, 4. All experiments were conducted on a 14/10 h light/dark cycle (lights on at 9 A.M. to 11 P.M.) with constant infrared illumination. All experiments were recorded at 25 Hz. Larvae were tracked continuously for 24–73 h, after which all larvae unresponsive to touch with a 10-μl pipette tip were presumed sick or dead and excluded from subsequent analysis. In the wild-type experiments we additionally excluded wells in which a bubble had formed under the plastic lid. The number of larvae excluded/used for each set of experiments is as follows: wild type, 164/288 (all exclusions were due to bubble artefacts); hypocretin, 0/180; melatonin, 0/96; PTZ, 44/96 (doses of 10, 15, and 30 mM were lethal to all tested larvae over 24 h). Following each experiment, larvae were euthanized with an overdose of 2-phenoxyethanol (Acros Organics).

### Fish lines

The term wild type refers to the AB x TUP LF zebrafish strain. This line was used for the wild-type experiments, as well as the melatonin and PTZ dose-response curves. *hcrtr* (ZFIN ID: hu2098; Yokogawa et al., 2007) experiments were conducted on embryos collected from heterozygous in-crosses, with larvae genotyped using KASP primers (LGC Genomics) post-tracking. KASP results were validated by comparison to PCR-based genotyping of samples from each KASP classified genotype.

### *hcrtr* genotyping

#### DNA extraction

Following each *hcrtr* experiment, each larva was euthanized in its well (as above) and DNA was extracted using HotSHOT DNA preparation (Truett et al., 2000). Larval samples were transferred to the individual wells of a 96-well PCR plate. Excess liquid was pipetted from each well before applying 50 μl of 1× base solution (1.25 M KOH and 10 mM EDTA in water). Plates were heat sealed and incubated at 95°C for 30 min, then cooled to room temperature before the addition of 50 μl of 1× neutralization solution (2 M Tris-HCl in water).

#### PCR

The following reaction mixture per sample was prepared on ice in a 96-well PCR plate: 18.3 μl PCR mix (2 mM MgCl_2_, 14 mM, pH 8.4, Tris-HCl, 68 mM KCl, 0.14% gelatin in water, autoclaved for 20 min, cooled to room temperature, chilled on ice, then we added: 1.8% 100 mg/ml BSA and 0.14% 100 mM d [A, C, G, T] TP), 0.5 μl of forward and reverse primers (20 μM), 5.5 μl water, 0.2 μl of Taq polymerase, and 3.0 μl of DNA. Next, each plate was heat sealed and placed into a thermocycler, set with the following program: 95°C, 5 min; 44 cycles: 95°C, 30 s; 57°C, 30 s; and 72°C, 45 s; then 72°C, 10 min and 10°C until collection. Finally, samples were mixed with 6× loading buffer [colorless buffer: Ficoll-400, 12.5 g, Tris-HCl (1 M, pH 7.4) – 5 ml, EDTA (0.5 M) – 10 ml, to 50 ml in pure water; heated to 65°C to dissolve, per 10 ml of colorless buffer 25 mg of both xylene cyanol and orange G were added, then diluted to 6×] and run on agarose gels (1% – 2%) with 4% GelRed (Biotium).

*hcrtr* forward primer: 5′-CCACCCGCTAAAATTCAAAAGCACTGCTAAC-3′; *hcrtr* reverse primer: 5′-CATCACAGACGGTGAACAGG-3′.

#### PCR information

PCR products were digested with Ddel at 37°C to produce a 170-bp band in the wild-type animals and 140- and 30-bp bands in *hcrtr* mutants.

#### KASP

KASP genotyping was conducted in white, low profile PCR plates on ice with six wells allocated 50:50 for positive and negative controls. The following reaction mixture was prepared per sample: 3.89 μl of 2× KASP reaction mix, 0.11 μl KASP primers, 1.0 μl water, and 3.0 μl DNA. Plates were then heat sealed and placed into a thermocycler with the following thermal cycling program: 94°C, 15 min; 10 cycles: 94°C, 20 s; 61–53°C (dropping 0.8°C per cycle), 60 s; 26 cycles: 94°C, 20 s; 53°C, 60 s, then 10°C until collection.

Following thermal cycling we used a fluorescence reader (Bio-Rad CFX96 Real-Time System) and Bio-Rad CFX Manager software (version 3.1) to automatically determine each sample’s genotype from a 2D scatter plot of fluorescence in each channel. From this scatter plot, outlying samples of unclear genotype were manually excluded from subsequent analysis.

#### KASP assay ID: 554-0090.1

KASP Flanking Sequence (alternative allele shown in square brackets, with a forward slash indicating a deletion in the alternative allele): 5′-ACCGCTGGTATGCGATCTGCCACCCGCTAAAATTCAAAAGCACTGCTAAA[A/T]GAGCCCGCAAGAGCATC GTGCTGATCTGGCTGGTGTCCTGCATCATGATG-3′.

### Pharmacology

0.15 M melatonin and 1 M PTZ (M5250 and P6500; Sigma) stock solutions were made in DMSO and sterile water, respectively. Behavioral testing concentrations for each compound were selected based on Rihel et al. (2010a). For behavior experiments, each animal in a well with 650 μl of fish water was dosed with 1.3 μl of either vehicle control or compound at 500× concentration, resulting in a 1 in 500 dilution and thus the desired testing concentration.

### Computing

#### Hardware

A desktop computer with 16 GB of RAM was used for most data analysis, figure production, and writing. For two-time intensive steps, hierarchical compression of full module sequences (Batch_Compress.m) and normalizing the behavioral motif counts (Batch_Grammar_Freq.m), data were run in parallel, with a worker for every animal, on the UCL Legion Cluster (Research Computing Services, UCL).

#### Software

All software used for data handling, analysis, and the production of figures is available at https://github.com/ghoshm/Structure_Paper. The codes are also available as Extended Data 1 and Extended Data 2.

10.1523/ENEURO.0408-19.2020.ed1 Extended Data 1 Supplementary Paper Code_Extended Data. Download Extended Data 1, ZIP file.

10.1523/ENEURO.0408-19.2020.ed2 Extended Data 2 Supplementary Legion Code_Extended Data. Download Extended Data 2, ZIP file.

#### Processing behavioral data

See Extended Data Figure 1-3 for a flow diagram describing behavioral data acquisition and analysis. All custom behavioral analysis software was written and run in MATLAB 2016b-2018a (MathWorks). The suffixes .m and .mat denote MATLAB code and MATLAB data files, respectively.

Behavioral data were recorded by subtracting subsequent pairs of frames from each other and determining the number of pixels that changed intensity within each well between each pair of frames, termed Δ pixels. To acquire behavior data, each Zebrabox was setup using ViewPoint’s ZEBRALAB software (version 3.22), which outputs a .xls and a .raw file (ViewPoint specific format) per experiment. Each behavior .xls file was reorganized into a .txt file using the function perl_batch_192.m (Jason Rihel). For each experiment a .txt metadata file assigning each animal to an experimental group, for example genotype, was manually produced. To replicate the previous analysis methodology, as in Extended Data Figure 1-1*D*, behavior and metadata .txt files were input to the function sleep_analysis2.m (Jason Rihel).

**Figure 1.**
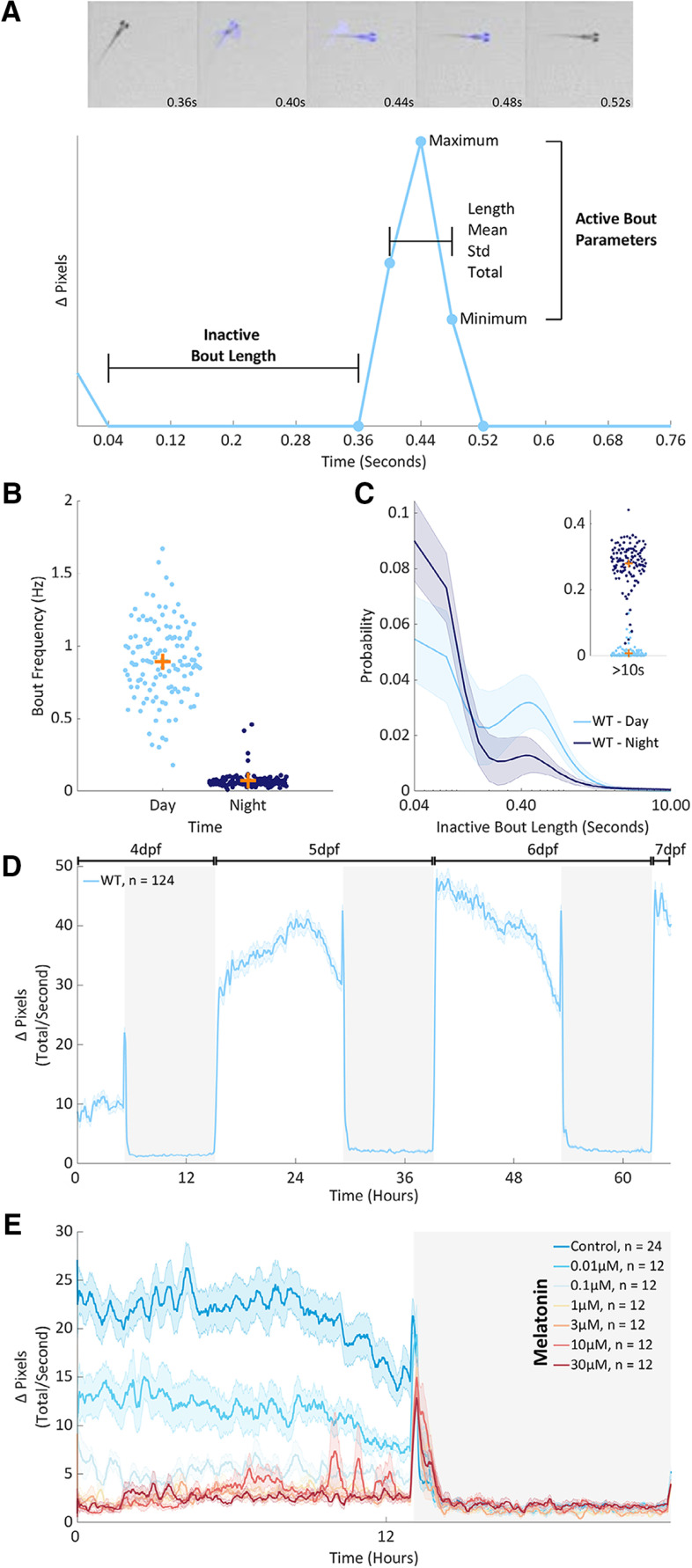
Behavior at scale. ***A***, top panel, Five consecutive frames from an individual well of a 96-well plate as a 6 dpf zebrafish larva performs a swim bout. Blue highlights pixels that change intensity between frames (Δ pixels). Lower panel. A Δ pixels time series from the larva above. Highlighted are the features that describe each active and inactive bout. ***B***, Mean bout frequency (Hz) recorded from individual larvae at 5 and 6 dpf during the day (light blue) and the night (dark blue). Each dot is 1 of 124 wild-type larvae. The orange crosses mark the population means. ***C***, The probability of observing different lengths of inactivity during the day (light blue) or the night (dark blue) at 5 and 6 dpf. Each larva’s data were fit by a pdf. Shown is a mean pdf (bold line) and SD (shaded surround) with a log scale on the *x*-axis cropped to 10 s. Inset, The total probability of inactive bout lengths longer than 10 s, per animal. ***D***, The mean activity of 124 wild-type larvae from 4 to 7 dpf, on a 14/10 h light/dark cycle. Data for each larva was summed into seconds and then smoothed with a 15-min running average. Shown is a summed and smoothed mean Δ pixels trace (bold line) and SEM (shaded surround). ***E***, Average activity across one day (white background) and night (dark background) for larvae dosed with either DMSO (control) or a range of melatonin doses immediately before tracking at 6 dpf. Data were summed and smoothed as in ***D***. The number of animals per condition is denoted as *n*. Extended Data Figures 1-1, 1-2, 1-3 support Figure 1.

10.1523/ENEURO.0408-19.2020.f1-1 Extended Data Figure 1-1 Behavioral set-up and analysis. ***A***, Schematic of our behavioral set-up. Note that aside from the computer, the set-up is fully enclosed. Not shown to scale. IR, infrared; LED, light emitting diode. ***B***, Normalized temporal correlation of active bout starts between 24 wild-type larva (6 dpf) across 24 h. Pairwise correlations were computed and then grouped into three groups: autocorrelation (orange); neighbor fish, defined as larva in adjacent wells, diagonals excluded (light blue); and distant, non-neighbor fish (dark blue). Data from each group are plotted as a mean (bold line) and SD (shaded surround). Note that the *y*-axis is cropped from 1, where autocorrelation peaks, to 0.1. ***C***, A fictive illustration of zebrafish behavior (blue line). Two minutes of data are shown divided by a black dashed vertical line. A 1-min binning approach would score both minutes as 20 s of activity and miss the 60-s period of inactivity in between. This latter loss leads to a discrepancy in the number of periods ≥60 s between the 1-min bin and 25-Hz methods (see ***D***). ***D***, The number of inactive periods ≥60 s for each of 124 wild-type animals is shown, as determined by both a 1-min bin and 25-Hz approach. Data are from each animal’s entire recording period (4–7 dpf). Data for each animal is shown as a pale blue line overlaid with a bold line showing the population mean and SD. Inset, The percentage of the 25-Hz counts detected by the 1-min bin method per animal. Each animal’s data are shown by a circle. An orange cross marks the population mean. ***E***, Average activity across one day (white background) and night (dark background) for larvae exposed to either H_2_O (control) or a range of PTZ doses immediately prior to tracking at 6 dpf. Data for each larva was summed into seconds and then smoothed with a 15-min running average. Shown is a mean summed and smoothed trace (bold line) and SEM (shaded surround); *n* denotes the number of animals per condition. Download Figure 1-1, TIF file.

To assess data on a frame by frame basis, each experiment's .raw file, which was output from Viewpoint’s Zebrabox, was exported within the ZEBRALAB software to thousands of .xls files. Each .xls file contained 50,000 rows and 21 columns, with data from any given well listed approximately every 192 rows, as the setup always assumes recordings are from two 96-well plates. This formatting is, however, only approximate as infrequently the well order is erroneously non-sequential; these rows were termed ordering errors. Each .xls file is formatted with 21 columns, of which three contain useful data: type, notes when ViewPoint defined data acquisition errors occurred; location, denotes which well the data came from; and data1, records the Δ pixel value from that well for that time point.

The function Vp_Extract.m was used to reformat the .xls files from each experiment to single frame by fish matrices, from which each animal’s behavior was quantified. Vp_Extract.m requires three inputs to be selected: a folder containing the .xls files; a .txt behavior file output from perl_batch_192.m; and a .txt metadata file. To ensure that each animal has the same number of frames, frames with ViewPoint defined errors or ordering errors (which are automatically detected by Vp_Extract.m) are discarded. A maximum Δ pixels value can be set and active bouts containing even a single frame with a higher Δ pixels value than this are set to zero for the entire duration of the bout. Here a maximum Δ pixels threshold of 200 was set. This value was determined from manual inspection of the dataset as well as by comparisons of these data to data recorded from plates with no animals in. Following this step, the maximum Δ pixels value observed was 165. Most Δ pixels values were between 3 (minimum observed value) and 30 (Extended Data Fig. 1-2*A*). Time periods during which water is being replenished are automatically detected and set to a Δ pixels value of zero. These time periods are noted and excluded from later analysis. The function outputs .mat files for subsequent analysis. Either single or multiple .mat files output from Vp_Extract.m were input to Vp_Analyse.m and Bout_Clustering.m.

**Figure 2.**
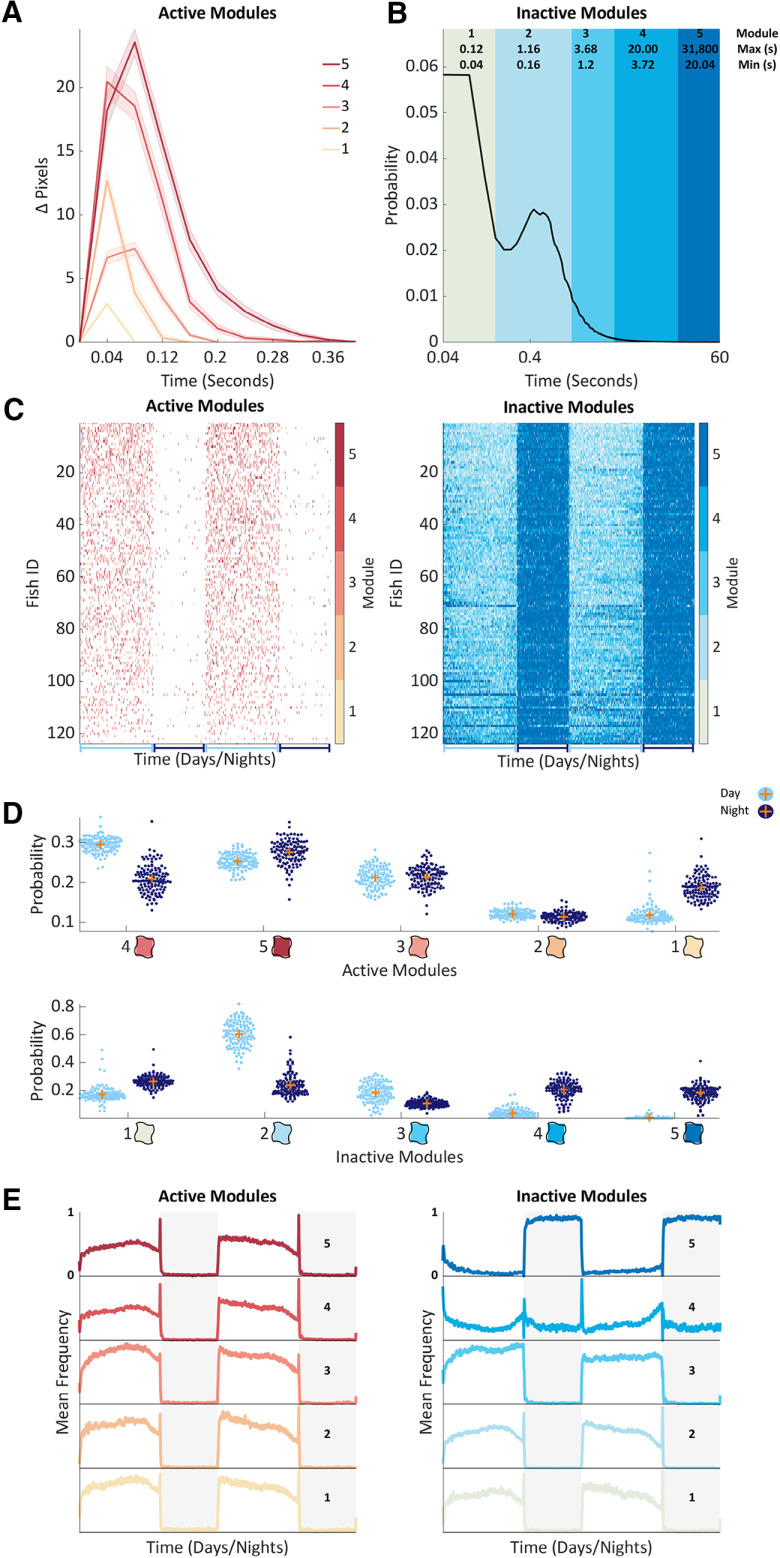
Unsupervised learning identifies contextual behavioral modules. ***A***, Average Δ pixels changes for each active module. Shown is the mean (bold line) and SEM (shaded surround) of 100 bouts randomly sampled from each module from one representative larva. Modules are numbered and colored by average module length across all animals, from shortest (1) to longest (5). ***B***, A probability density curve showing the distribution of inactive bout lengths in seconds, on a log *x*-axis cropped to 60 s. Modules are numbered and colored from shortest (1) to longest (5) mean length (see legend for each module’s minimum and maximum bout length). ***C***, Matrices showing the active (left) or inactive (right) module assignment of every frame (*x*-axis) for each of 124 wild-type larvae (*y*-axis) across the 14-h days (light blue underlines) and 10-h nights (dark blue underlines) from 5 to 6 dpf. Larvae were sorted by total number of active modules from highest (top) to lowest (bottom). Modules are colored according to the adjacent colormaps. ***D***, Average active (upper) and inactive (lower) module probability during day (light blue) and night (dark blue) 5 and 6 of development. Each of 124 wild-type animals is shown as a dot and orange crosses mark the population means. Active modules are sorted by mean day probability from highest to lowest (left to right). Inactive modules are sorted by mean length from shortest to longest (left to right). The blobs correspond to the color used for each module in other figures. ***E***, The mean frequency of each active (left) and inactive (right) module across days 5 and 6 of development. Shown is a mean smoothed with a 15-min running average, rescaled to 0–1. Days are shown with a white background, nights with a dark background. Modules are sorted from shortest to longest (lower to upper panels). Extended Data Figures 2-1, 2-2 support Figure 2.

10.1523/ENEURO.0408-19.2020.f1-2 Extended Data Figure 1-2 Bout Features. ***A***, Bout feature distributions during the day (light blue) and the night (dark blue). For the probability curves, each animal’s data were fit with a pdf. Shown is a mean pdf (bold line) and SD (shaded surround) with a log scale on the *x*-axis. For the scatter plots, each larva’s mean value across the days or nights (5–6 dpf) is shown as a light blue (day) or dark blue circle (night). An orange cross marks each population’s mean. Of the pdfs, only the mean day and night active bout total and inactive bout length pdfs were consistently significantly different across three independent experiments (*p* < 0.01; two-sample Kolmogorov–Smirnov test); *n* = 124 wild-type larvae. ***B***, Melatonin bout feature means. A mean was taken per animal per feature, and day or night (6 dpf). Shown is a population mean and SEM during the day (white background) and the night (grey background). Control, DMSO; *n* = 24 controls then *n* = 12 per dose. ***C***, PTZ bout feature means, as in ***B***. Control, H_2_O; *n* = 24 controls then *n* = 10 (2.5 mM), *n* = 9 (5 mM), and *n* = 9 (7.5 mM). ***D***, *hcrtr* bout feature means as in ***B***, for days (white background) and nights (grey background) 5–6 post fertilization. *hcrtr*^-/-^ mutants had significantly lower mean values compared to both *hcrtr*^+/+^ and *hcrtr*^-/+^ for the following active bout features: length, SD and total (*p* < 0.05 for all comparisons, Dunn–Sidak corrected four-way ANOVA, adjusted for the following factors: day/night, development, and experimental repeat). No features differed significantly between *hcrtr*^-/+^ and *hcrtr*^+/+^; *n* = 39, 102, and 39; for WT, *hcrtr*^+/+^; Het, *hcrtr*^-/+^; and Hom, *hcrtr*^-/-^, respectively. Download Figure 1-2, TIF file.

**Figure 3.**
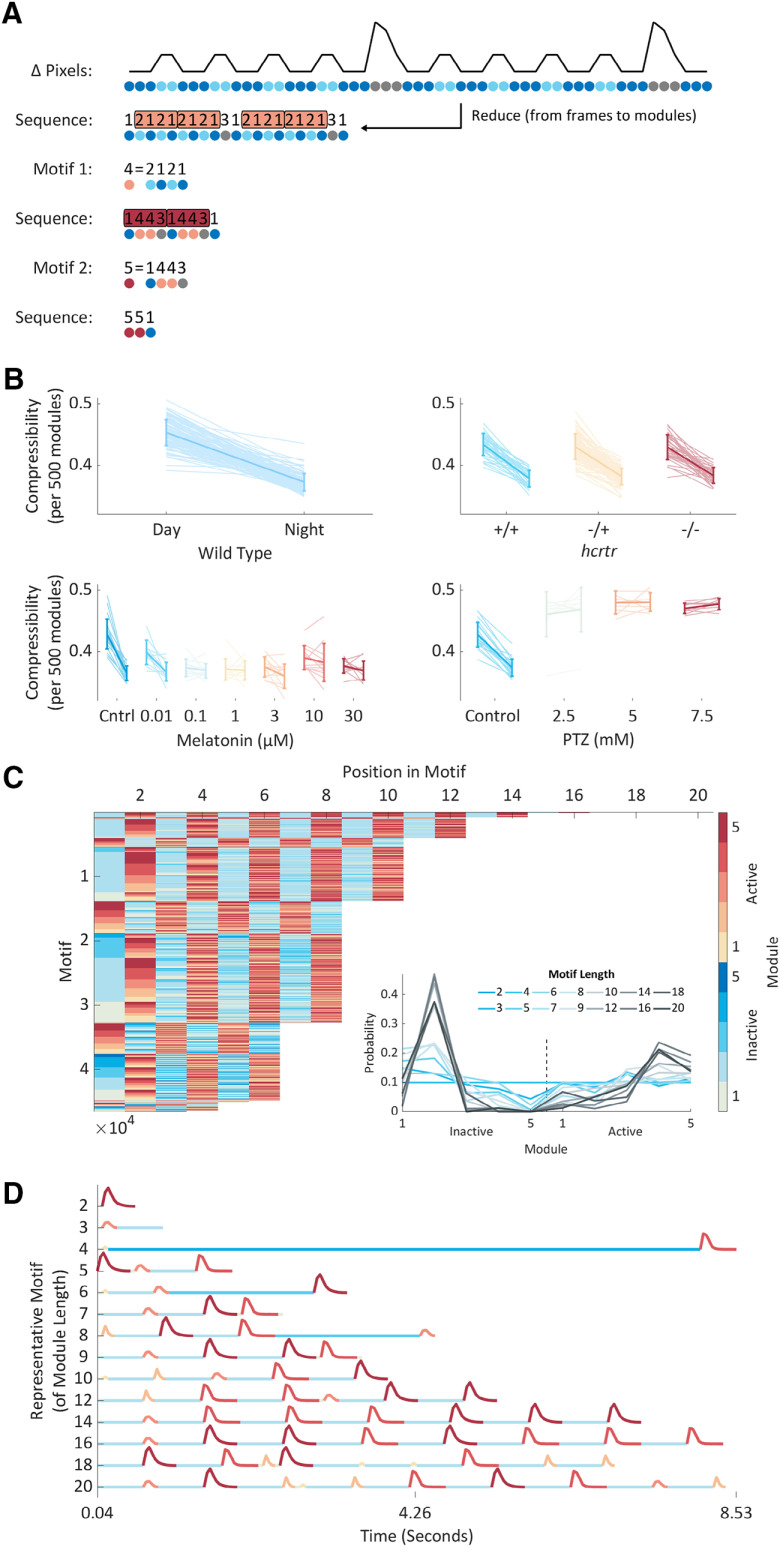
Hierarchical compression reveals structure in zebrafish behavior. ***A***, Compression explained using fictive data. Top to bottom, From Δ pixels data (black trace), we classified both active and inactive behaviors into modules (colored circles). From modular behavioral sequences, we identified motifs (sequences of modules) using a compression algorithm. Compression iteratively identifies motifs (shown as boxes) by replacing them with new symbols until no more motifs can be identified and the sequence is maximally compressed. ***B***, Each panel shows how compressibility, calculated from 500 module blocks, varies in different behavioral contexts. Each pale line shows an individual fish’s mean compressibility during the day and the night. The darker overlay shows a population day and night mean ± SD. In the wild-type data, compressibility is higher during the day than the night (*p* < 10^−158^) and increases from day/night 5–6 (*p* < 10^−4^), findings consistent across triplicate experiments. Melatonin decreases (*p* < 10^−10^), while PTZ increases compressibility (*p* < 10^−8^). There is no effect of *hcrtr* genotype on compressibility. Statistics are two-way or four-way ANOVA. ***C***, All 46,554 unique motifs (*y*-axis) identified by compressing data from all animals. Each motif’s module sequence is shown, with the modules colored according to the colormap on the right. Motifs are sorted by length and then sequentially by module. Motifs range in length from 2 to 20 modules long. Inset, For each motif length, the probability of observing each inactive or active module. ***D***, Each motif in the library consists of an alternating sequence of Δ pixels changes and pauses (active and inactive modules). A representative motif of each module length is shown with each module colored according to the colormap in ***C***. Representative motifs were chosen by determining every motif’s distribution of modules and then for each observed module length, selecting the motif closest to the average module distribution (see ***C***, inset). Extended Data Figure 3-1 supports Figure 3.

10.1523/ENEURO.0408-19.2020.f1-3 Extended Data Figure 1-3 Analysis framework. Flow diagram depicting the steps of our analysis framework. Data are output from our behavioral set-up (ViewPoint) in the form of a .xls file. perl_batch_192.m organizes these data to a .txt format. Experiment metadata (e.g., animal genotypes) are supplied in the form of a .txt file. The 1-min bin method uses sleep_analysis2.m to produce figures and statistics from these two .txt files. The 25-Hz method exports .raw data from ViewPoint to produce .xls files. Vp_Extract.m reorganizes these, using .txt data, to a .mat file which can be input to either Vp_Analyse.m or Bout_Clustering.m. Vp_Analyse.m produces figures and statistics. Bout_Clustering.m uses the clustering function gmm_sample_ea.m to assign data to modules, produce figures, and calculate statistics, Bout_Clustering.m’s output can be input to Bout_Transitions.m, which compresses full modular sequences by calling Batch_Compress.m and Batch_Grammar_Freq.m. The motifs identified from this approach can be input to Batch_Transitions_Hours.m which compresses 500 module chunks and uses Batch_Grammar_Freq.m to count motif occurrences per hour. With the exception of the 1-min bin method (sleep_analysis2.m), two example figures are shown for each figure producing step. All code can be run locally, although for speed several steps (indicated in green) are best run on a cluster computer. Download Figure 1-3, TIF file.

Vp_Analyse.m was used to compare general activity levels and bout features across time and between groups. The function has two options. The first allows for specific days and nights of interest to be cropped from the data. The second determines how experimental repeats are handled, treating the data as either a single merged dataset or as separate datasets. In the latter case, each experimental repeat is plotted with the same color scheme as the first experiment, with progressive shading for each repeat. Additionally, the *N*-way ANOVA comparisons include a repeat factor, which can be used to determine whether results are consistent across experimental repeats. Vp_Analyse.m outputs two statistics results structures: twa, *N*-way ANOVA comparison results; and kw, two-sample Kolmogorov–Smirnov test results. Vp_Analyse.m outputs figures showing each group’s activity (Fig. 1*D*,*E*) and bout features (Extended Data Fig. 1-2) over time.

The script Bout_Clustering.m was used to cluster all active and inactive bouts into behavioral modules, as well as to compare the resultant modules. To cluster the data an evidence accumulation approach is used (Fred and Jain, 2002, 2005) implemented by the custom MATLAB function gmm_sample_ea.m. Bout_Clustering.m produces figures (Extended Data Fig. 2-1) and statistically compares the modules. The MATLAB workspace output from Bout_Clustering.m can be input to either Bout_Transitions.m or Bout_Transitions_Hours.m.

10.1523/ENEURO.0408-19.2020.f2-1 Extended Data Figure 2-1 Evidence accumulation-based clustering. ***A***, left, Scree plot showing the percentage of variance explained by each principal component from the active bout data. The first three principal components, the knee point of the curve, were kept for subsequent analysis. The colors of these points refer to the right panel. Right, Each of the three retained component’s coefficients for the different active bout parameters is shown. ***B***, The active bouts within each module were fit by Gaussian distributions. Each active bout is shown in a 3D space of PC1, PC2, and probability. Each bout is numbered and colored by its module assignment. ***C***, Evidence accumulation (E.A.) matrix for the 40,000 active probe points (matrix dimensions are thus 40,000 by 40,000). A higher E.A. index indicates a higher frequency of pairwise occurrences in the same cluster across 200 Gaussian mixture models. This matrix was clustered hierarchically, and a maximum lifetime cut was made to determine the final number of modules. The dendrogram above shows all 40,000 leaves and is colored by mean module length from shortest (lightest) to longest (darkest) as in other figures. 500. Evidence accumulation matrix for the inactive bouts. Download Figure 2-1, TIF file.

The function gmm_sample_ea.m clusters data using an evidence accumulation approach (Fred and Jain, 2002, 2005) through which the results of multiple Gaussian mixture models are combined to generate an aggregate solution. This process is executed through the following six steps. First, a sample of “probe points” are randomly sampled from the data. The number of probe points to sample is user defined. Second, values of K and sample sizes are uniformly sampled from user set ranges. The values of K are used to set the number of mixture components for each mixture model. The sample sizes determine the number of points, randomly sampled from the data that each mixture model is fit to. Third, a Gaussian mixture model is iteratively fit to the sampled data with K components. Each probe point is assigned to the component with the highest corresponding posterior probability and evidence is accumulated on the probe points; evidence is defined as pairwise co-occurrences in the same component. Fourthly, the evidence accumulation matrix is hierarchically clustered, and the final number of clusters is determined by using the maximum differentiated linkage distance to cut the resultant dendrogram. The linkage metric used is a user-defined option. Fifthly, the clusters are normalized for size by randomly sampling the number of points in the smallest cluster, from each cluster. Finally, all data points are assigned to these final size normalized clusters using the mode cluster assignment of the k-nearest neighbors, with k being user defined.

The script Bout_Transitions.m takes the MATLAB workspace output from Bout_Clustering.m as an input and compresses each animal’s full module sequence to generate a library of behavioral motifs. The number of occurrences of each motif are counted and normalized by comparison to paired shuffled data. Finally, a supervised learning algorithm is applied to identify context specific behavioral motifs. For two-time intensive steps, hierarchical compression of full module sequences (Batch_Compress.m) and normalizing the behavioral motif counts (Batch_Grammar_Freq.m), data were manually copied (via MobaXterm, Personal Edition v10.5) to UCL Legion Cluster (Research Computing Services, UCL) and processed in parallel with a worker for every fish. MATLAB code for hierarchical compression is described in Gomez-Marin et al. (2016). MATLAB code for submitting these jobs to Legion, analyzing data and retrieving results is available at https://github.com/ghoshm/Legion_Code. Ultimately, Bout_Transitions.m outputs a library of behavioral motifs and motif-related figures (Fig. 3).

**Figure 4.**
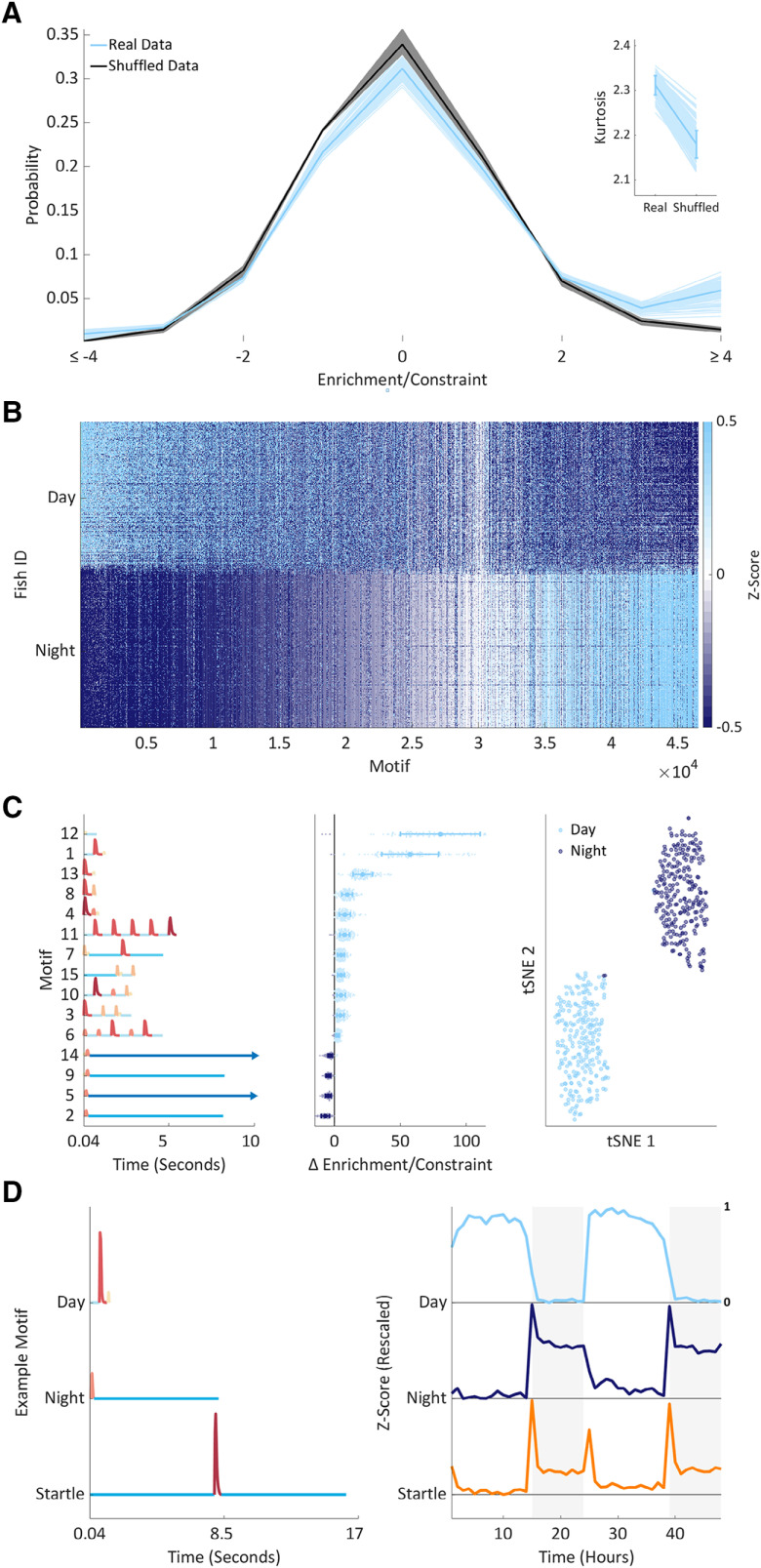
Supervised learning identifies contextual behavioral motifs. ***A***, pdfs showing the probability of observing motifs at different enrichment/constraint scores rounded to whole numbers and summed at values above or below ±4 for ease of visualization. Each wild-type animal is depicted by a single pale blue (real data) and 10 black (shuffled data) lines; overlaid in bold are mean pdfs. The inset shows that the kurtosis of the real data are higher than the shuffled data (*p* < 10^−271^; two-way ANOVA, real vs shuffled data, no significant interaction with experimental repeat factor). Each larva is shown as a pale line; overlaid is a population mean and SD. ***B***, Enrichment/constraint scores for all 46,554 motifs (*x*-axis) for each fish during day/night 5 and 6 of development (*y*-axis). To emphasize structure, motifs are sorted in both axes, first by their average day/night difference (from day to night enriched left to right), then separately day and night by larva. Finally, each motif’s enrichment/constraint score is Z-scored to aid visualization. ***C***, left, The 15 day/night mRMR motifs module sequences are shown numbered by the order in which they were selected by the algorithm. Motifs are sorted by day minus night enrichment/constraint score (middle). The long pauses at the end of motifs 5 and 14 are cropped at 10 s (arrows). Middle, For each selected motif (*y*-axis), ordered as in the left panel, each wild-type animal’s (124 in total) day minus night enrichment/constraint score (*x*-axis) is shown as a dot. Values above zero are colored light blue; below zero are dark blue. Overlaid is a population mean and SD per motif. Right, A tSNE embedding of the 15-dimensional motif data (middle) into a two-dimensional space. Each circle represents a single day (light blue) or night (dark blue) sample. ***D***, Representative motif temporal dynamics; shown are motifs 1 (day) and 2 (night) from ***C***, as well as a startle-like motif. Left, Each motif’s module sequence. Right, Each motif’s mean enrichment/constraint score each hour, rescaled to 0–1. Extended Data Figure 4-1 supports Figure 4.

The script Bout_Transitions_Hours.m compresses blocks of 500 modules for statistical comparisons, uses the motif library from Bout_Transitions.m to count the occurrence of each motif every hour, normalizes these counts to paired shuffled data and finally uses supervised learning to identify hour specific behavioral motifs. As with Bout_Transitions.m behavioral motifs are normalized, via Batch_Grammar_Freq.m, using UCL Legion Cluster. Bout_Transitions_Hours.m outputs figures (Fig. 4*D*) and statistics.

**Table 1:** Wild-type motif classifier performance Download Figure 4-1, TIF file.

Comparison	Motifs (number)	Cv error (%)	Cv error Std	Mc error (%)	Mc EP	RM error (%)	RM error Std
Wild type							
Day/night	15	0.20	0.63	50.0	2.25	9.25	2.50
Day 5/day 6	93	20.2	9.60	50.0	3.18	45.8	4.09
Night 5/night 6	85	19.8	8.09	50.0	3.18	48.3	6.00
Day hours							
•09-10	102	6.39	1.23	7.14	0.44	7.67	0.26
•10-11	1	7.37	0.31	7.14	0.44	7.18	0.10
•11-12	5	7.20	0.23	7.14	0.44	7.15	0.03
•12-13	9	7.06	0.34	7.14	0.44	7.19	0.08
•13-14	1	7.14	0.12	7.14	0.44	7.14	0
•14-15	1	7.14	0.12	7.14	0.44	7.14	0
•15-16	1	7.11	0.14	7.14	0.44	7.14	0
•16-17	1	7.09	0.15	7.14	0.44	7.14	0
•17-18	1	7.14	0.12	7.14	0.44	7.14	0
•18-19	1	7.14	0.12	7.14	0.44	7.14	0
•19-20	1	7.14	0.12	7.14	0.44	7.14	0
•20-21	3	7.11	0.27	7.14	0.44	7.15	0.01
•21-22	1	7.14	0.12	7.14	0.44	7.14	0
•22-23	1	7.14	0.12	7.14	0.44	7.15	0.03
Night hours							
•23-24	23	0.69	0.47	10.0	0.60	6.57	1.23
•24–01	177	9.84	1.83	10.0	0.60	11.6	0.78
•01–02	5	9.92	0.51	10.0	0.60	10.0	0.05
•02-03	88	9.72	1.18	10.0	0.60	10.4	0.31
•03-04	1	10.0	0.17	10.0	0.60	10.0	0.04
•04-05	22	9.92	0.47	10.0	0.60	10.1	0.07
•05-06	1	10.0	0.17	10.0	0.60	10.0	0
•06-07	1	10.0	0.17	10.0	0.60	10.0	0.02
•07-08	3	9.84	0.34	10.0	0.60	10.0	0.01
•08-09	1	10.0	0.17	10.0	0.60	10.0	0.01
Morning/evening	229	33.2	2.32	50.0	0.85	44.7	0.96
Early/late night	26	36.4	2.18	50.0	1.00	43.4	1.55

A table showing the performance of each wild-type motif classifier. Each classifier sought to separate the data shown in the comparison column, e.g., day/night. For the hourly comparisons, each hour was compared with data from all other hours grouped together. For each comparison, 250 motifs were chosen by mRMR, then a smaller number were retained (see motifs column) based on classification error curves (Extended Data Fig. 4-1*A*). Cv, 10-fold cross validated; Std, SD across the 10 folds; Mc, majority class classifier; EP, SE of proportion; RM, classifiers built from random motif subsets.

**Table 2:** *hcrtr* and pharmacological classifier performance Download Figure 4-1, TIF file.

Comparison	Motifs (number)	Cv error (%)	Cv error Std	Mc error (%)	Mc EP	RM error (%)	RM error Std
*hcrtr*							
Day and night							
•WT/Het	173	25.5	6.77	27.7	1.88	39.0	1.93
•WT/Hom	83	24.7	6.07	50.0	2.83	47.8	3.66
•Het/Hom	235	24.7	3.76	27.7	1.88	38.6	1.83
Day							
•WT/Het	80	19.5	9.60	27.7	2.66	37.9	1.08
•WT/Hom	195	16.7	7.50	50.0	4.00	48.7	4.22
•Het/Hom	55	22.7	7.02	27.7	2.66	33.8	2.01
Night							
•WT/Het	79	16.3	6.38	27.7	2.66	37.1	7.17
•WT/Hom	53	12.8	9.58	50.0	4.00	52.3	6.16
•Het/Hom	76	16.0	7.27	27.7	2.66	36.0	5.05
Melatonin (day)							
•Control	40	0	0	25.0	4.42	16.4	3.67
•0.01μM	89	1.39	4.52	16.7	4.39	30.0	15.2
•0.1μM	192	1.39	4.52	16.7	4.39	20.5	18.0
•1μM	132	2.78	6.02	16.7	4.39	29.5	8.67
•3μM	97	0	0	16.7	4.39	48.6	11.2
•10μM	250	2.78	6.02	16.7	4.39	20.0	9.40
•30μM	133	0	0	16.7	4.39	32.2	7.89
PTZ (day)							
•Control	26	0	0	46.2	6.91	15.8	6.43
•2.5 mM	55	0	0	35.7	9.06	42.1	11.4
•5 mM	162	0	0	32.1	8.83	34.3	18.0
•7.5 mM	104	0	0	32.1	8.83	49.9	14.7

A table showing the performance of each classifier. Each classifier sought to separate the data shown in the comparison column, e.g., *hcrtr*^+/+^ (WT) and *hcrtr*^−/+^ (Het). For the pharmacological comparisons, each condition was compared with the rest of the conditions grouped together, aside from the control data which was excluded. For each comparison, 250 motifs were chosen by mRMR, then a smaller number were retained (see motifs column) based on classification error curves (see Extended Data Fig. 4-1*A*). Cv, 10-fold cross validated; Std, SD across the 10 folds; Mc, majority class classifier; EP, SE of proportion; RM, classifiers built from random motif subsets; WT, *hcrtr*^+/+^; Het, *hcrtr*^−/+^; Hom, *hcrtr*^−/−^.

**Table 3:** Module classifier performance Download Figure 4-1, TIF file.

Comparison	Modules (number)	Cv error (%)	Cv error Std	Mc error (%)	Mc EP
Wild type							
•Day/Night	10	1.61	1.29	50.0	2.25
•Day 5/Day 6	8	21.0	6.53	50.0	3.18
•Night 5/Night 6	1	35.5	9.71	50.0	3.18
*hcrtr*							
Day and night							
•WT/Het	1	27.7	0.77	27.7	1.88
•WT/Hom	10	45.8	10.9	50.0	2.83
•Het/Hom	8	27.5	1.12	27.7	1.88
Day							
•WT/Het	1	27.7	1.46	27.7	2.66
•WT/Hom	1	40.4	12.5	50.0	4.00
•Het/Hom	3	27.3	2.35	27.7	2.66
Night							
•WT/Het	1	27.7	1.46	27.7	2.66
•WT/Hom	1	47.4	10.9	50.0	4.00
•Het/Hom	10	27.0	1.72	27.7	2.66
Melatonin (day)							
•Control	3	8.33	8.69	25.0	4.42
•0.01μM	10	2.78	6.02	16.7	4.39
•0.1μM	2	16.7	4.52	16.7	4.39
•1μM	1	18.1	7.74	16.7	4.39
•3μM	1	16.8	8.67	16.7	4.39
•10μM	1	16.8	4.52	16.7	4.39
•30μM	1	16.8	4.52	16.7	4.39
PTZ (day)							
•Control	1	1.92	5.27	46.2	6.91
•2.5 mM	1	17.9	17.6	35.7	9.06
•5 mM	1	28.6	22.3	32.1	8.83
•7.5 mM	10	20.0	26.1	32.1	8.83

A table showing the performance of each module classifier. Each classifier sought to separate the data shown in the comparison column, e.g., wild type, day/night. For each comparison, all 10 modules were sequentially chosen by the mRMR algorithm, then a smaller subset was retained (see module column) based on classification error curves. Cv, 10-fold cross validated; Std, SD across the 10 folds; Mc, majority class classifier; EP, SE of proportion.

Processed behavioral data are available at: https://zenodo.org/record/3344770#.XYYwYyhKiUk. Raw data are available upon request.

### Behavioral data analysis

#### Δ Pixels

At the acquisition stage, Δ pixels data were filtered by the software (ViewPoint) such that each frame for a given well was scored as either zero or higher. In the absence of movement within a well, and hence no pixels changing intensity, Δ pixels values of zero were recorded. These periods were termed inactive bouts and were defined as any single or consecutive frames with Δ pixels values equal to zero. The length of each inactive bout was used as a descriptive feature. When there were movements within a well, Δ pixels values greater than zero were recorded. These periods were termed active bouts and were defined as any single or consecutive frames with Δ pixels values greater than zero. Six features were used to describe each active bout: length, mean, SD, total, minimum, and maximum. These features, as well as the number of active bouts, percentage of time spent active and total Δ pixels activity, were compared between conditions, e.g., day and night and dose of drug, in two ways using the function Vp_Analyse.m.

To compare the distribution of values for each feature between conditions, a probability density function (pdf) was fit to each animal’s data and the mean shape of each condition’s pdf was compared using a two-sample Kolmogorov–Smirnov test (Extended Data Fig. 1-2*A*). To compare each feature’s average values between conditions, mean values were taken from each animal, and *N*-way ANOVA was computed. The following factors, when relevant, were included and full interaction terms were calculated: condition, e.g., mutant and wild type; time, e.g., day and night; development, defined as a consecutive day and night; and experimental repeat, i.e., which experimental repeat a datapoint came from. For experiments with multiple repeats, the lack of an interaction effect between the comparison of interest and experimental repeat factor was considered as evidence of a consistent result.

Note that our analysis relies on periods of inactivity registering Δ pixels values of zero. Consequently, to be compatible with our analysis code, other data may require post-recording filtering.

#### Clustering

To cluster the bouts, the script Bout_Clustering.m was used. First, matrices of bouts by features were constructed (active matrix, 30,900,018 × 6; inactive matrix, 30,900,418 × 1). To prepare the active data for clustering, each animal’s data were individually normalized by calculating *z* scores using Equation 1, which illustrates how every bout (i) from each animal (f) was normalized by first subtracting the mean of this animal’s bout features (${\bar{x}}_{f}$) from the bout and then dividing by the SD of each bout feature for this animal σ_f_:

(1) $$Z_{i}=\frac{x_{i}-{\bar{x}}_{f}}{\sigma_{\text{f}}}.$$

Active bout features across all animals were then centered by subtracting each feature’s mean value from every bout, and principal component analysis (PCA) was used to reduce the data to three dimensions, the knee point of the scree plot, which together explain 97.5% of the variance (Extended Data Fig. 2-1*A*).

Next, the active and inactive bouts were separately clustered using an evidence accumulation-based approach (Fred and Jain, 2002, 2005) implemented by the function gmm_sample_ea.m. First, 40,000 probe points were randomly sampled from the data. Next, for 200 iterations, another group of points were randomly sampled and fit with a Gaussian mixture model with a random number of clusters. For each iteration, these two parameters varied uniformly in the following ranges: the number of points sampled, 40,000–100,000; the number of clusters fit, 2–20. Each mixture model was fit using MATLAB’s fitgmdist function (MATLAB, Statistics and Machine Learning Toolbox) with full, regularized, independent covariance matrices and initialized using the k-means++ algorithm (Arthur and Vassilvitskii, 2007). Each mixture model was fit five times and the one with the largest log-likelihood was retained. Once each model had been fit, each probe point was assigned to the component with the largest posterior probability, and evidence in the form of pairwise occurrence in the same cluster was accumulated on the probe points. Once the 200 mixture models had been fit, average link clustering was applied to the evidence accumulation matrix and the final number of clusters determined based on maximum cluster lifetime. Next, the resultant clusters were normalized for size by randomly selecting the number of points in the smallest cluster from each cluster (5983 active, 614 inactive bouts). Finally, all points were assigned to the size normalized clusters using the mode cluster assignment of the 50 nearest neighbors for every point.

#### Hierarchical compression

Clustering reduced each animal’s behavior to a non-repetitive sequence of active and inactive bouts, termed modules. On average this reduced each wild-type sequence length by 96%, from 6,308,514 frames to 236,636 modules, easing the computational demands of compressing these sequences.

To compress modular sequences, an offline compressive heuristic (Nevill-Manning and Witten, 2000) was used (Eq. 2). At each iteration (i) of the algorithm, the most compressive motif was defined as the motif which made the most savings, a balance between the length of the motif (*W*) and the number of times it occurred in the sequence (*N*), which also considered the combined cost of adding a new motif to the dictionary (*W* + 1) and of introducing a new symbol into the sequence (+N) at every occurrence of this motif in the sequence:

(2) $$Savings_{i}=WN-\left(W+1+N\right).$$

The overall compressibility of a given input sequence was calculated by summing these savings across all iterations and dividing this total by the length of the original input sequence (in modules). This process resulted in a compressibility metric that ranged from 0 to 1 (low-high compressibility). To reduce computational time, motifs of a maximum of 10 modules long were sought, although the hierarchical nature of the algorithm enabled the identification of longer motifs through nesting. To generate the common motif library, the motifs obtained from compression of every animal’s full module sequence (Batch_Compress.m) were merged, and then all unique motifs were kept (Bout_Transitions.m). To generate sets of paired control sequences for every animal, each animal’s module sequence was divided into sequential day and night or hourly segments and the modules within each of these windows was shuffled 10 times, maintaining the active/inactive transition structure (Bout_Transitions.m). As compressibility varies non-linearly with uncompressed sequence length (Extended Data Fig. 3-1*B*), to enable comparisons between samples with different numbers of modules, non-overlapping blocks 500 modules long were compressed (Bout_Transitions_Hours.m).

#### Supervised motif selection

To identify both which and how many motifs were required to distinguish between behavioral contexts (e.g., day and night), the following approach was executed by the function Batch_Grammar_Freq.m. First, the number of occurrences of every motif from the common motif library was counted in every real and shuffled modular sequence. Next, to calculate enrichment/constraint scores for every motif, the deviation of the real from shuffled counts, as well as the deviation of each shuffle from the other shuffles, was calculated (Eq. 3). For a given animal and time window, e.g., day or night, the mean number of times motif (i) was counted in the shuffled data (${\bar{s}}_{i}$) was subtracted from the real number of counts ($x_{i}$) and divided by the SD of the shuffled counts ($\sigma_{si}$):

(3) $$Z_{i}=\frac{x_{i}-{\bar{s}}_{i}}{\sigma_{si}}.$$

For example, for a given animal and time window, if a motif was used 10 times in the real data and a mean of 4.5 times in the 10 sets of paired shuffled data (SD 1.58), enrichment/constraint would equal 3.48:

$$3.48=\frac{10-4.5}{1.58}.$$

When comparing the shuffled data to itself, each shuffle (now $x_{i}$) was excluded from ${\bar{s}}_{i}$ and $\sigma_{si}$. Infinite values occurred when there was no SD in the $\sigma_{si}$ counts and thus $\sigma_{si}$ equaled zero. To facilitate subsequent working, infinite values were replaced with a constant value of √(1 + number of shuffled counts) = √(11) = ±3.32. Note that in the real data, infinite values constituted only 2.2% of all enrichment/constraint scores.

**Figure 5.**
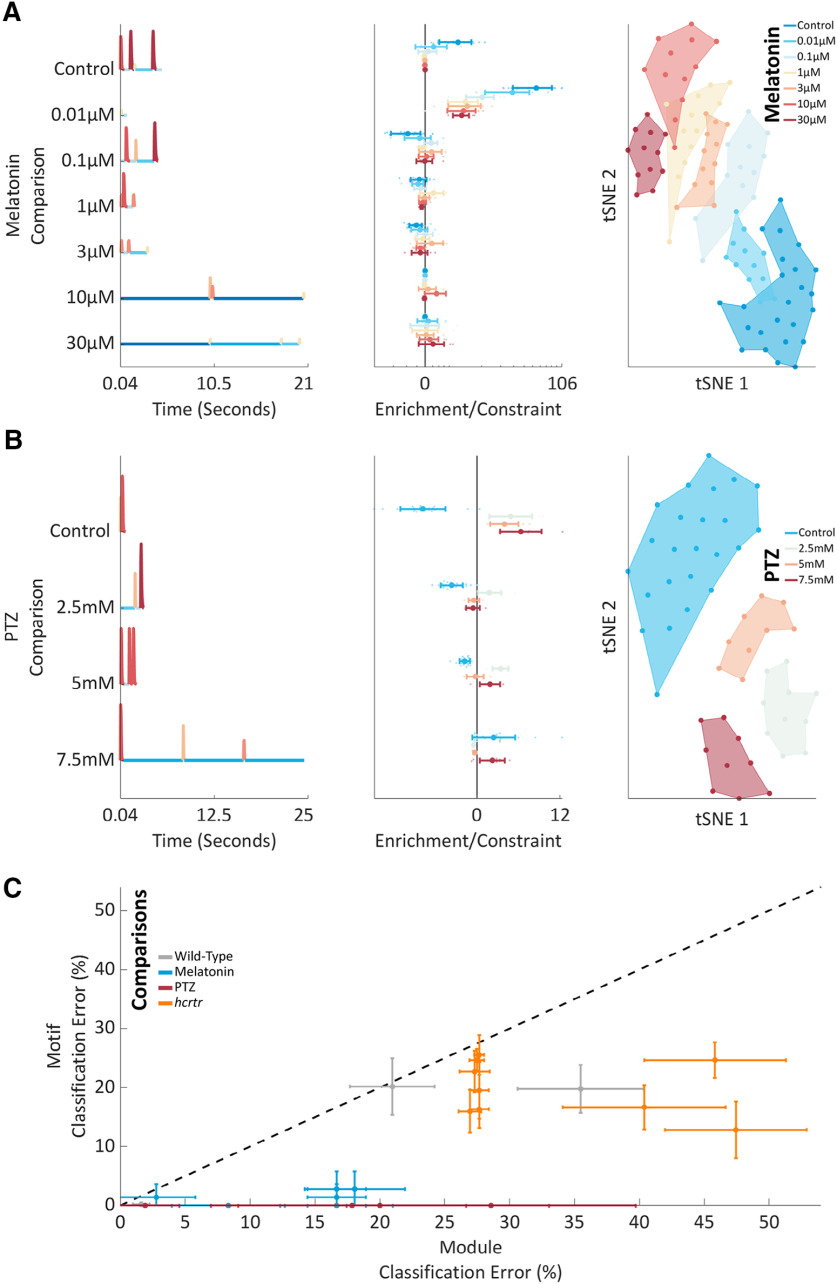
Pharmacological behavioral motifs. ***A***, left, Module sequences for the single best motif for each melatonin comparison. Modules are colored as elsewhere. Middle: for each dose’s single best motif, see left panel *y*-axis for dose, enrichment/constraint scores are shown for every dose on a log *x*-axis. Each animal is shown as a dot, with a mean ± std overlaid per dose. Right, A two-dimensional tSNE embedding from a space of 912 unique motifs. Each animal is shown as a single dot underlaid by a shaded boundary encompassing all animals in each condition. ***B***, left, Module sequences for the single best motif for each PTZ comparison. To highlight a seizure specific motif, the control motif and corresponding enrichment/constraint score shown is mRMR motif 2, not 1, for this comparison. Modules are colored as elsewhere. Middle, For each dose’s single best motif, enrichment/constraint scores are shown for every dose on a linear *x*-axis. Each animal is shown as a dot, with a mean and SD overlaid per dose. Right, A two-dimensional tSNE embedding from a space of 338 unique motifs. Each animal is shown as a single dot underlaid by a shaded boundary encompassing all animals in each condition. ***C***, Each classifier’s classification error (%) is shown in terms of modules (*x*-axis) and motifs (*y*-axis). Data are shown as mean and SD from 10-fold cross validation. Classifiers are colored by experimental dataset (see Legend). For reference, y = x is shown as a broken black line. Data below this line demonstrates superior performance of the motif classifiers.

For any given comparison, motif library enrichment/constraint scores for the relevant animals were formatted into a matrix of samples by motifs (Fig. 4*B*). Scores for each motif (column) were normalized by subtracting each column’s mean score and dividing by each column’s SD. A supervised feature selection algorithm (Peng et al., 2005) was applied to these matrices to select the top 250 maximally relevant and minimally redundant (mRMR) motifs. For each comparison we defined the first motif chosen by mRMR as the best motif. To determine the best motifs among test conditions, we excluded control data. For example, to determine the best 0.01 μM melatonin motif, we compared this group’s data to all larvae dosed with melatonin at other doses (Fig. 5*A*). For completeness, we still plot the control enrichment/constraint scores for these motifs.

To determine how many of the 250 motifs chosen by mRMR were necessary for accurate classification, linear discriminant analysis classifiers were trained on data using 10-fold cross validation as sequential mRMR motifs were added, and classification error mean and SD were calculated. The MATLAB function fitcdiscr (Statistics and Machine Learning Toolbox) was used to implement these steps. Finally, to determine how many motifs were necessary for a given comparison, classification error curves were smoothed with a running average three motifs wide and the number of motifs at which the minimum classification error occurred was identified (Extended Data Fig. 4-1*A*). To evaluate classifier performance, the results of each classifier were compared with a majority class classifier whose performance depended on the ratio of samples of each class. For example, in a dataset with two labels at a ratio of 0.1:0.9, the majority class classifier would consistently assign the latter label and achieve a classification error of 10% (±SE of proportion). Additionally, we compared each classifiers performance to a set of 10 classifiers built using the same number of motifs, although randomly selected. For example, for a classifier which achieved its minimal classification error using 15 motifs, we randomly selected 15 motifs and trained classifiers as above. We repeated this process 10 times per classifier and report the error and SD across these 10 repeats.

## Results

### Behavior at scale

Larval zebrafish behavior consists of an alternating sequence of movements and pauses, termed bouts, that are organized at sub-second timescales. To capture this structure from high-throughput, long-timescale experiments, we used a 96-well plate set-up with a single larva housed in each well (Extended Data Fig. 1-1*A*) and as a proxy for movement recorded the number of pixels that changed intensity within each well between successive pairs of frames, a metric we term Δ pixels. We built on previous work using this set-up (for review, see Barlow and Rihel, 2017; Oikonomou and Prober, 2017) by analyzing Δ pixels data at 25 Hz, rather than in 1-min bins. When recorded in this way, Δ pixels data are an alternating sequence of positive values representing movement magnitude and zeros representing periods of inactivity (Fig. 1*A*;Movie 1). We defined active bouts as any single or consecutive frames with non-zero Δ pixels values and described each bout using several features including the mean and SD of Δ pixels values across the bout (Fig. 1*A*). We defined inactive bouts as any single or consecutive frames with zero Δ pixels values and described each inactive bout using its length (Fig. 1*A*). We found no correlation of active bout initiation between larvae in adjacent wells of the 96-well plate (Extended Data Fig. 1-1*B*), suggesting no interactions among larvae.

Movie 1. High-throughput behavioral tracking. A video of 96, 6 dpf zebrafish larvae swimming in our rig. The last 1 s of each larva’s Δ pixels data is plotted over each well. This video was filmed at 25 Hz and is played back in real time. 10.1523/ENEURO.0408-19.2020.video.1

Using this approach, we first assessed the behavior of wild-type larvae across a 14/10 h day/night cycle (Extended Data Fig. 1-2*A*). During the day, wild-type larvae initiated bouts at a mean frequency of 0.89 Hz (Fig. 1*B*), a rate consistent with other assays (Kim et al., 2017), and tended to use short, sub-second long inactive bouts (Fig. 1*C*). During the night, larvae displayed a mean bout frequency of only 0.07 Hz (Fig. 1*B*) and used longer inactive bouts, on the order of seconds to minutes (Fig. 1*C*). Together, these differences resulted in a diurnal pattern of activity (Fig. 1*D*). These results are broadly consistent with those from analysis of binned Δ pixels data (Barlow and Rihel, 2017; Oikonomou and Prober, 2017), with the addition of sub-second resolution and an increase in accuracy, as determined by intra-fish comparisons between the methods (Extended Data Fig. 1-1*C*,*D*).

Next, we extended our approach to examine the behavioral effects of pharmacological and genetic manipulations. Melatonin, which is strongly hypnotic in zebrafish (Rihel et al., 2010a), dose dependently decreased larval activity (Fig. 1*E*) by decreasing the number, magnitude, and length of active bouts and by inducing longer inactive bouts (Extended Data Fig. 1-2*B*). The epileptogenic drug PTZ (Extended Data Fig. 1-1*E*) altered both active and inactive bout parameters (Extended Data Fig. 1-2*C*), eliciting on average longer, lower amplitude active bouts and longer inactive bouts during the day. Finally, homozygous *hcrtr*^−/−^ mutants had only subtle differences in active bout structure, with shorter mean active bout length and lower active bout total and SD, compared with both wild-type *hcrtr*^+/+^ and heterozygous *hcrtr*^-/+^ siblings, which did not differ from one another by any metrics (Extended Data Fig. 1-2*D*).

Collectively, these results quantitatively demonstrate the advantages of assessing Δ pixels data on a frame by frame basis and provide insight into the behavior of wild-type zebrafish larvae across the day/night cycle as well as those subject to pharmacological or genetic manipulations.

### Module usage varies with behavioral context

Recent work has demonstrated that larval activity can be classified using unsupervised learning into 13 distinct bout types that represent different swimming movements (Marques et al., 2018). A full description of larval behavior, however, requires quantification of both the movements and pauses that they execute. Thus, we sought to determine whether distinct active or inactive bout types, which we termed modules, were identifiable from our data, and if module usage depended on behavioral context.

To address these questions, we separately clustered the active and inactive bouts (combined across experiments a total of 30,900,018 active and 30,900,418 inactive bouts) using an evidence accumulation-based clustering algorithm (see Materials and Methods). In brief, 200 Gaussian mixture models were built from each data set, then the results of these models were combined to generate aggregate solutions. This clustering method identified five active and five inactive modules (Fig. 2*A*,*B*; Extended Data Fig. 2-1), which we separately labeled from 1 to 5 from the shortest to longest mean bout length. The active modules, which formed discrete peaks in bout feature space (Extended Data Fig. 2-1*B*), corresponded to different shapes of Δ pixel changes in terms of amplitude and length (Fig. 2*A*; Extended Data Fig. 2-2*A*), while the inactive modules consisted of different lengths of inactivity (Fig. 2*B*; Extended Data Fig. 2-2*A*). The shortest inactive module (module 1) had a mean length of 0.06 s and ranged from a minimum of 0.04 s (our sampling limit) to a maximum of 0.12 s. In contrast, the longest inactive module (module 5) had a mean length of 96 s and covered a huge range of values from a minimum of 20 s to a maximum of 8.8 h.

10.1523/ENEURO.0408-19.2020.f2-2 Extended Data Figure 2-2 Behavioral modules. ***A***, pdfs for each bout feature by module. All features are shown on a log *x*-axis. The legend panel indicates each module’s color. ***B***, Melatonin module probabilities during 6 dpf day (upper panels) and night (lower panels) for both the active (left) and inactive (right) modules. Shown is a mean and SEM for each group, colored according to the legend. Active modules are sorted from highest to lowest by average wild-type day probability, based upon wild-type data in Figure 2*D*. Inactive modules are sorted by increasing mean length. Control, DMSO; *n* = 24 controls then *n* = 12 per dose. ***C***, PTZ data as in ***B***, with H_2_O (control); *n* = 24 controls then *n* = 10 (2.5 mM), *n* = 9 (5 mM), and *n* = 9 (7.5 mM). ***D***, *hcrtr* data as in ***B***, with mean values across 5 and 6 dpf. No module probabilities differed significantly among genotypes (full four-way ANOVA, with the following factors: genotype, day/night, development, and experimental repeat); *n* = 39, 102, and 39; for WT, *hcrtr*^+/+^; Het, *hcrtr*^-/+^; and Hom, *hcrtr*^-/-^, respectively. Download Figure 2-2, TIF file.

10.1523/ENEURO.0408-19.2020.f3-1 Extended Data Figure 3-1 Hierarchical compression metrics. ***A***, The compressibility (*y*-axis) of the real wild-type data is higher than the paired shuffled data (*p* < 10^−15^, two-way ANOVA, real vs shuffled data, no significant interaction with experimental repeat factor). Each animal’s data are shown as a pale blue line. Overlaid is a mean and SD. Inset, The mean difference in compressibility between each larva’s real and shuffled data. Each larva is shown by a circle, and the orange cross marks the mean. ***B***, The compressibility (*y*-axis) of the real wild-type data varies non-linearly with uncompressed sequence length. Each larva (of 124) is shown as a dot. ***C***, The number of motifs (*y*-axis) identified from compressing each wild-type animal’s real and paired shuffled data. Each animal’s data are shown as a pale blue line. Overlaid is a mean and SD. Inset, The mean intra-fish difference in the number of identified motifs. Each larva is shown by a circle, and the orange cross marks the mean. ***D***, Motif length (*x*-axis) and usage probability (*y*-axis) across the entire real (blue) and 10 shuffled datasets (black). Note that each shuffled dataset is plotted independently. For each motif length a grey line joins the real and mean shuffled value. ***E***, Each panel shows how Δ compressibility varies in different behavioral contexts. Each pale line shows an individual larva’s average Δ compressibility during the day and the night. The darker overlay shows a population day and night mean and SD. ***F***, Δ Compressibility of 500 module blocks for each wild-type larva, averaged into 1-h time points. Each pale blue line shows 1 of 124 larvae. Line breaks occur when a larva had <500 modules within a given hour. The darker blue overlay shows the mean and SD of these data every hour. Shown are days (white background) and nights (dark background) 5 and 6 of development. Download Figure 3-1, TIF file.

10.1523/ENEURO.0408-19.2020.f4-1 Extended Data Figure 4-1 Motif classifier performance. ***A***, Classification error (%) from linear classifiers separating wild-type day and night behavior using motif enrichment/constraint scores as sequential mRMR motifs from 1 to 250 are added (*x*-axis). The average error is shown in light blue. Overlaid in darker blue is a running average three motifs wide. The broken black lines show the minimum of the smoothed data to be at 15 motifs, where the classification error is 0.2%. ***B***, Wild-type temporal classifier performance. Real classifiers (color) are shown as a mean and SD from 10-fold cross validation. Majority class classifiers (grey) are shown as value and SE of proportion. Each classifier’s data are listed on the *x*-axis. D, day; N, night; M/E, morning/evening; E/LN, early/late night. The number of motifs chosen for each classification and exact values for each classifier are detailed in Table 1. ***C***, *hcrtr*, Melatonin and PTZ classifier performance. Real classifiers (color) are shown as a mean and SD from 10-fold cross validation. Majority class classifiers (grey) are shown as value and SE of proportion. Each classifier’s data are listed on the *x*-axis. For *hcrtr* comparisons, grouped classifiers as well as separate day (light blue underline) and night (dark blue underline) classifiers are shown. For melatonin and PTZ, only day data were compared. Classifier details can be found in Table 2. Download Figure 4-1, TIF file.

To examine how module usage varied across time, we represented each larva’s behavior as an alternating sequence of active and inactive modules (Fig. 2*C*; Movie 2). In the wild-type data, module usage varied with time of day (Fig. 2*D*). For example, the probability of observing inactive module 2, which consists of typical day pause lengths (0.16–1.16 s), was on average 0.6 during the day and only 0.24 during the night, when inactive modules 1, 4, and 5 became more likely (Fig. 2*D*). To reveal finer-grain temporal dynamics, we also examined each module’s mean frequency over time (Fig. 2*E*). In general, both the active and the short inactive modules had high frequencies during the day, peaking at the light/dark transition as the larvae responded to the sudden change in illumination. In contrast, the only module with a peak in frequency at the dark-to-light transition was inactive module 4 (3.72–20 s), which also had an increased frequency approaching the light-to-dark transition. Together, these results reveal that zebrafish employ different bout types in a time of day/night dependent manner.

Movie 2. Behavioral modules. A video of 96, 6 dpf zebrafish larvae swimming in our rig. The last 1 s of each larva’s Δ pixels data is plotted over each well, with each active and inactive bout colored according to its module assignment. This video was filmed at 25 Hz and is played back in real time. 10.1523/ENEURO.0408-19.2020.video.2

Next, we examined the impact of pharmacological and genetic manipulations on bout type usage. Larvae dosed with melatonin showed a shift toward using shorter active modules and longer inactive modules (Extended Data Fig. 2-2*B*). In PTZ-dosed larvae, there were also shifts in active module probability. Particularly notable was the complete exclusion of active module 1 in 27 of the 28 (96.4%) PTZ-dosed larvae, while control larvae used this module with 0.12 probability during the day and 0.22 during the night (Extended Data Fig. 2-2*C*). These shifts likely reflect the chaotic, seizure-like swimming observed in PTZ-treated larvae (Baraban et al., 2005), although no single active module clearly captured these behavioral seizures. PTZ also increased the probability of the shortest inactive (module 1) as well as the two longest inactive modules (modules 4 and 5), the latter of which are likely to correspond to the interictal bouts of inactivity associated with seizures (Extended Data Fig. 2-2*C*). Conversely, *hcrtr* mutants exhibited no differences in either active or inactive module probabilities compared with their wild-type siblings (Extended Data Fig. 2-2*D*), demonstrating that bout type usage is similar between these mutants and wild-type animals across the day/night cycle.

Collectively, these results reveal that zebrafish behavior in this assay can be described by five types of active and five types of inactive modules, the usage of which varies with behavioral context. Interestingly, in many contexts, both active and inactive module probabilities were shifted, suggesting that these module types may co-vary, perhaps by being arranged into recurrent sequences.

### Hierarchical compression reveals structure in zebrafish behavior

From a set of behavioral modules, an animal could structure their behavior in a range of ways. At one end of this spectrum, successive modules could be organized completely randomly, such that prior modules exert no influence on future module selection. At the other end, module selection could be fully deterministic with a particular module always following another. Rather than being fixed, however, it is likely that animals adapt their behavioral structure in response to changing internal or external states. We sought to map the structure of zebrafish behavior in different contexts by examining the presence and organization of module sequences, which could provide insight into the mechanisms governing behavior. To do this, we used a compression algorithm (Nevill-Manning and Witten, 2000) as Gomez-Marin et al. (2016) used to discover structure in *Caenorhabditis elegans* postural data. When applied to our dataset (Fig. 3*A*), this algorithm iteratively identified motifs from each larva’s modular sequence and returned two outputs, compressibility, a measure of each larva’s behavioral structure, and a library of identified recurrent module sequences, termed motifs.

To quantify the structure of zebrafish behavior, we first compressed every animal’s full modular sequence, which in wild-type animals were on average 236,636 modules long across 70 h. To determine whether the resultant compression values indicated more structure than would be expected based on either the distribution or the transition structure of the active-to-inactive modules, we compared each larva’s compressibility to that of 10 sets of paired shuffled data. All wild-type larvae were more compressive than their paired shuffled data, demonstrating that their behavior is more structured than expected from modular probabilities alone (Extended Data Fig. 3-1*A*). Compressibility, however, varies non-linearly with input sequence length, as longer sequences will be more likely to contain motifs (Extended Data Fig. 3-1*B*). Thus, to enable comparisons between samples with different numbers of modules, we compressed non-overlapping 500 module blocks of sequence per larva. This approach revealed that compressibility was higher during the day than the night (Fig. 3*B*). To determine whether these differences were primarily due to the presence of behavioral motifs or instead were a consequence of differences in module distribution, we also compared the difference in compressibility (Δ compressibility) between each animal’s real and shuffled data. This approach revealed that the compressibility difference between the day and the night is predominantly due to differences in module distribution (Extended Data Fig. 3-1*E*). To reveal finer-grain temporal changes in compressibility, we plotted Δ compressibility across time (Extended Data Fig. 3-1*F*). This approach revealed peaks at the light-to-dark transitions in the evenings, consistent with this stimulus eliciting stereotyped behavioral sequences (Burgess and Granato, 2007; Emran et al., 2010).

Next, we used compressibility to assess how our pharmacological and genetic manipulations altered the structure of larval behavior. We found that melatonin decreased day compressibility to night-time levels (Fig. 3*B*), a difference primarily due to shifts in module distribution (Extended Data Fig. 3-1*E*). PTZ increased compressibility to a constant day/night value (Fig. 3*B*). PTZ, however, reduced Δ compressibility (Extended Data Fig. 3-1*E*), indicating that changes in module distribution, rather than motif usage, are the dominant driver of PTZ-induced behavioral changes. Importantly, these drug-induced changes in compressibility do not simply reflect overall activity levels. For example, PTZ-exposed larvae are less active than controls during the day and more active during the night (Extended Data Fig. 1-1*E*) but have consistently higher compressibility (Fig. 3*B*). Finally, in *hcrtr* mutants, we found no differences in either compressibility or Δ compressibility, suggesting that *hcrtr* mutant behavior is structured similarly to wild-type animals (Fig. 3*B*).

Overall, our compressibility results demonstrate that larval zebrafish behavior is structured and that the amount of structure varies with behavioral context. Our Δ compressibility results suggest that such variation predominantly arises from altered module, rather than motif, usage.

To gain insight into the behavioral sequences larvae deploy, we then studied the motifs identified by the compression algorithm. Compression of the real modular sequences identified a mean of 1901 motifs per animal (Extended Data Fig. 3-1*C*). Interestingly, compression of the real data almost always identified slightly fewer motifs than the shuffled data (Extended Data Fig. 3-1*C*). This suggests that the motifs identified from the real data were used more frequently than those in the shuffled data and therefore likely reflect enriched behavioral sequences. Merging the motifs identified across all animals generated a library of 46,554 unique behavioral motifs (Fig. 3*C*). In terms of raw Δ pixels data, each motif represented an approximately repeated pattern of movements and pauses of varying length (Fig. 3*D*). Motifs in the library ranged from two to 20 modules long with a median length of eight modules and spanned timescales from approximately 0.1 s to 11.3 min with a median length of 3.84 s. In the real data, longer motifs tended to be used more frequently than in the shuffled data (Extended Data Fig. 3-1*D*), demonstrating the likely behavioral relevance of these sequences. Motifs of different module lengths used distinct sub-sets of modules (Fig. 3*C*). For example, motifs comprised of longer module sequences had a lower probability of using long inactive modules. Together, these results reveal the varied timescales at which zebrafish larvae organize their behavior and suggest the presence of structure governing the arrangement of modules into motifs.

### Behavioral motif usage is time dependent

The large number of motifs in our library led us to hypothesize that each may be used in specific behavioral contexts. To test this hypothesis, we counted the number of times each larva or set of paired shuffled data used each motif within each time frame (e.g., day or night) and then normalized these counts by calculating the deviation of the real from shuffled counts as well as the deviation of each shuffle from the other shuffles, a metric we termed enrichment/constraint. Overall, we found that enrichment/constraint scores from our real data were more prone to extreme positive (enriched) and negative (constrained) values than the shuffled data (Fig. 4*A*), suggesting that a minority of behavioral motifs were used more or less frequently than would be expected by chance.

To test whether these extremes occurred in particular contexts, we first compared motif usage between the day and the night in wild-type larvae by generating a matrix of enrichment/constraint scores (Fig. 4*B*). To distil the most salient motifs from this and other contextual matrices, we used a three-step approach. First, we used the mRMR algorithm (Peng et al., 2005) to rank the motifs from most to least salient. Second, we trained linear discriminant analysis classifiers using 10-fold cross validation as we iteratively increased the number of input motifs from most to least salient (e.g., motif 1, motif 1 and 2, motif 1 – *n*). Finally, we selected the subset of motifs which achieved the lowest classification error between groups in each context. To determine how accurately these motif subsets could distinguish between behavioral contexts, we compared each classifier’s performance to that of a majority class classifier, which performed as well as the ratio of samples between the two contexts. For example, in the day versus night classification, a majority class classifier would have an error rate of 50% (±SE of proportion), as each larva contributes an equal number of days and nights to the enrichment/constraint matrix. Additionally, to demonstrate the salience of the motifs selected by the mRMR algorithm, we compared each classifier’s performance to a set of 10 classifiers built using the same number of motifs, although randomly selected. For example, for a classifier that achieved its minimal classification error using 50 motifs, we randomly selected 50 motifs from the library and built a classifier. For each comparison we repeated this process 10 times.

Applying this algorithm to wild-type data revealed changes in motif usage across multiple timescales (Extended Data Fig. 4-1*B*). We found that only 15 motifs were required to classify day-specific and night-specific behavior with only a 0.2% (±0.63% SD) classification error, compared with a majority class classifier with 50% error and random 15-motif subsets with a mean error of 9.25% (Fig. 4*C*; Table 1). The day-enriched motifs consisted of high amplitude movements interspersed with short pauses, while the night enriched motifs contained low amplitude movements and long pauses (Fig. 4*C*). Next, we examined how motif usage changed over time by comparing consecutive days and nights (5–6 dpf). In both day 5 versus day 6 and night 5 versus night 6 comparisons, the classifiers achieved roughly 20% error using 93 and 85 motifs, respectively (Table 1). Thus, motif usage shifted over just 24 h, although these changes were far less prominent than those between the day and night. To study whether motif usage varied at finer timescales, we first divided the day into morning/evening and the night into early/late periods. In each case, the mRMR algorithm performed better than the control classifiers (morning/evening: 33%, early/late night: 36%) although the relatively high classification errors suggest that motif selection did not vary strongly across each day or night (Table 1). Consistent with this conclusion, classifiers attempting to delineate each hour from every other mostly failed to outperform their control classifiers (Table 1). The two notable exceptions were the hour following each lighting transition, where this approach achieved good classification performance (Table 1) and identified startle-like motifs. We interpret these motifs as startle-like as they consist of long pauses interrupted by high-amplitude movements and primarily occur at the lighting transitions (Fig. 4*D*), stimuli known to elicit startle behaviors in larval zebrafish (Burgess and Granato, 2007; Emran et al., 2010).

Together, these results demonstrate that motif usage varied between the day and the night, but aside from the lighting transitions, was relatively consistent within these periods.

### Dose-dependent and dose-specific behavioral motifs

Finally, we hypothesized that behavioral motif usage would vary dose dependently across concentrations of melatonin and PTZ, providing insight into the mechanisms by which these compounds exert their behavioral effects. Motif dose dependency would suggest a continuously modulated underlying process, which might arise for example if the fraction of bound receptors relates to neuronal activity modulation. Alternatively, motifs enriched at only specific doses would suggest discrete effects on neuronal circuitry.

Applying the mRMR algorithm to our pharmacological data revealed both dose-dependent and dose-specific modulation of motif usage. We found that each melatonin dose could be separated from the others using 40–250 motifs with only 0–2.78% classification error (Fig. 5*A*; Table 2). Focusing on just the best motif for each comparison, we observed both dose dependency as well as dose specificity. For example, comparing controls to all melatonin-dosed larvae identified a dose-dependent motif that consisted of large magnitude movements and short pauses, whose enrichment/constraint score decreased with increasing melatonin concentration (Fig. 5*A*). Conversely, the best 10 μM motif, two long pauses broken by a small active bout sequence, showed dose specificity being enriched at only 3 and 10 μM doses (Fig. 5*A*). When applied to the PTZ data, our approach performed even more accurately, achieving perfect classification (0% error) between all conditions (Fig. 5*B*; Table 2). Furthermore, in PTZ-dosed larvae we observed enrichment for motifs highly constrained in wild-type larvae, highlighting the usage of motifs beyond the normal wild-type repertoire, such as those corresponding to behavioral seizures (Fig. 5*B*).

Next, we tested whether our motif subset approach could detect *hcrtr* mutant phenotypes that were not easily captured by other methods. For example, based on human and rodent literature, where loss of hypocretin is associated with narcolepsy (Lin et al., 1999) and prior zebrafish literature (Elbaz et al., 2012), we expected abnormal transitions between active and inactive bouts. We found reasonable performance when discriminating between *hcrtr*^+/+^ and *hcrtr*^−/−^ during both the day (16.7 ± 7.5% error with 195 motifs) and night (12.8 ± 9.6% error with 53 motifs) but weaker performance when distinguishing between *hcrtr*^+/+^ and *hcrtr*^−/+^, as expected for a haplosufficient gene (Extended Data Fig. 4-1*C*; Table 2). Thus, homozygous loss of *hcrtr* impacts motif usage enough to allow for successful classification of *hcrtr*^−/−^ mutants, although no single *hcrtr*^−/−^ motifs with large differences in enrichment/constraint scores compared with wild-type siblings were particularly evident.

Collectively, these results demonstrate that behavioral motifs are used context dependently and reveal how motif subsets can parse subtle differences in motif usage between behavioral contexts. However, does motif analysis provide additional discriminatory power over module selection, which also varies between behavioral contexts? To assess this, we compared the performance of each motif classifier to paired module classifiers built from matrices of module probabilities. All of the motif classifiers achieved better performance than their module pairs (Fig. 5*C*; Table 3), demonstrating both the phenotyping value of the motifs and their importance in the structure of larval behavior.

## Discussion

Here, we developed and applied computational tools to describe high-throughput, long-timescale behavioral data in terms of behavioral units (modules) and sequences of modules (motifs) organized across sub-second to day-long timescales.

### Low-dimensional representations of behavior

Low-dimensional representations of behavior, such as the Δ pixels metric employed here, result in a loss of information, for example, direction of movement or posture. However, such metrics do facilitate screening approaches and/or long-timescale tracking and in these contexts have provided biological insight into the molecular targets of small molecules (Rihel et al., 2010a) and genetics of aging (Churgin et al., 2017). Our work builds on previous long-timescale studies of behavior by assessing sub-second resolution Δ pixels data across multiple days and nights. This improved resolution enabled the segmentation and parameterization of individual active and inactive bouts from our data, revealing how larvae adapt their behavior across the day/night cycle and how behavior is impacted by small molecules.

Future work should aim to extend our assay by recording more detailed behavioral measures. Indeed, a recent study using centroid tracking in 96-well plates revealed that larvae show a day/night location preference within the well and furthermore uncovered a mutant with a difference in this metric (Thyme et al., 2019), demonstrating that even within the confined space of a 96-well plate, location is an informative metric to record. It is likely that even more detailed behavioral measures, like eye and tail angles, will yield additional insights, for example enabling the exploration of rapid-eye-movement sleep in zebrafish larvae, as done in reptiles (Shein-Idelson et al., 2016). Such metrics could be extracted by skeletonization or even through the use of an autoencoder applied to the raw video frames from each well (Johnson et al., 2016). Once such high dimensional data had been assigned to modules, our compression and motif enrichment/constraint approach could be applied in the same manner as here.

### Modular descriptions of behavior

A key idea in ethology is that behavior consists of stereotyped modules arranged into motifs (Lashley, 1951; Tinbergen, 2010). While early studies described behavior in this manner through manual observations (Dawkins and Dawkins, 1976), recent advances in machine vision and learning have automated these processes (Todd et al., 2017). For example, in zebrafish larvae, recent work used unsupervised learning to uncover a locomotor repertoire of 13 swim types including slow forward swims and faster escape swims (Marques et al., 2018), although inactive bouts were not considered. From our dataset, we identified five active and five inactive modules, which, respectively, describe swim bouts of different amplitudes (Fig. 2*A*) and periods of inactivity of varied length (Fig. 2*B*). Interestingly, all modules were used with reasonably high and similar probability by all wild-type animals (Fig. 2*D*), demonstrating that these modules represent a set of common larval behaviors. Furthermore, the temporal (Fig. 2*E*) and pharmacological (Extended Data Fig. 2-2*B*,*C*) shifts in these probabilities illustrates that module usage can be flexibly reorganized depending on behavioral context (Wiltschko et al., 2015).

To discretize our bouts into modules, we first extracted hand-engineered features from each bout (Fig. 1*A*) and then applied an evidence accumulation-based clustering algorithm (Fred and Jain, 2002, 2005). While our results demonstrate the relevance and utility of these modules in describing larval behavior, it is possible that our approach missed rare bout types. Consequently, future work should build on our bout classification by exploring the benefits of including additional features, the use of alternative clustering algorithms and our assumption of stereotypy, i.e., that all bouts can be fit into a module (Berman, 2018). An alternative direction would be to produce a mapping between our active modules and those identified from analysis of larval posture (Marques et al., 2018). Bridging this gap could facilitate behavioral screening approaches, for example, by using data from our set-up to prioritize pharmacological compounds or mutants for postural analysis.

### Quantifying structure in behavior

In some contexts, it is beneficial for animals to execute coordinated patterns of behavior. For example, to efficiently search an environment zebrafish larvae will execute organized sequences of left and right turns (Dunn et al., 2016). In other contexts, more random behavior will be advantageous, such as when escaping from a predator (Maye et al., 2007). Quantifying structure in behavior thus provides insight into the overarching strategy being employed in particular contexts. Alterations in behavioral structure can also manifest clinically, for example in Autism Spectrum Disorder, a defining feature of which is increased behavioral stereotypy (American Psychiatric Association, 2013). Consequently, compression would be a relevant and likely informative metric to record in animal models or even human cases for such conditions.

To quantify structure in larval zebrafish behavior in different contexts, we inputted each larva’s modular sequence to a compression algorithm. We found that wild-type behavior was more compressive during the day than the night (Fig. 3*B*). This echoes recent work in *Drosophila* that revealed higher temporal predictability during the day than the night as well as in females (Fulcher and Jones, 2017). A likely explanation for these findings comes from work in *C. elegans* (Gomez-Marin et al., 2016) that demonstrated that animals who transition slowly between modules, as both zebrafish (Fig. 1*B*) and *Drosophila* do at night (Geissmann et al., 2019), tend to be less compressive. This may suggest that the underlying mechanisms controlling longer-timescale behaviors are less precise than those controlling fast behavioral sequences.

For future efforts applying compression to behavioral data, there are two avenues left to explore, what compression heuristic to use and how to compress data from multiple animals. Following the work of Gomez-Marin et al. (2016), we defined the best motif at any iteration as the most compressive, which represents a balance between the motif’s length and frequency. While this metric generally leads to the best compression (Nevill-Manning and Witten, 2000), alternative measures, such as frequency or length may capture other aspects of behavior. The second avenue relates to comparisons between animals. Here, each animal was compressed individually, identifying motifs that were later grouped into a common library. While computationally tractable, this approach prevents certain comparisons across animals, for example identifying the most compressive motif across all larvae. This issue could be solved by compressing a single sequence containing all of the animals’ modular sequences joined end to end, with spacers to prevent interanimal motifs. Compressing this long sequence would, however, be computationally demanding.

Compressing and merging the identified motifs across all animals generated a library of 46,554 unique motifs (Fig. 3*C*), each of which described an alternating sequence of movements and pauses (Fig. 3*D*). Motifs ranged from 0.1 s to 11.3 min in length, revealing the range of timescales at which larval behavior is organized. We cannot, however, rule out the existence of longer timescale motifs in larval behavior as computational demands limited our search to motifs 10 modules long (although the algorithm’s hierarchical approach enabled the identification of motifs up to 20 modules long). Thus, future work should aim to extend our approach to explore the full range of timescales at which larval behavior is organized by systematically varying this parameter.

### Contextual behavioral motifs

Finally, by distilling salient subsets of motifs from our library, we demonstrated that motif usage was context dependent and highlighted the discriminatory power of motif subsets, which were capable of distinguishing between day/night behavior and even between small changes in compound dose. Comparing motif usage across the day/night cycle identified a set of highly night specific motifs (Fig. 4*C*), which may represent sleep behaviors. One way in which future studies could address this possibility would be to deprive larvae of these motifs throughout the night, for example, by using a closed-loop paradigm (Geissmann et al., 2019), and observing the impact on larval behavior the following day. In relation to the PTZ data, comparing seizure motifs across epileptogenic compounds and mutants with spontaneous seizures could suggest clues as to their underlying mechanism (Kokel et al., 2010; Rihel et al., 2010a). For example, seizures with similar motif usage patterns may originate in the same brain area or impact awareness in the same manner. This hypothesis could be tested by generating whole-brain activity maps (Randlett et al., 2015) across conditions, with the aim of identifying common and unique neuronal correlates.

Given the amenability of larval zebrafish to high-throughput behavioral screening (Rihel and Ghosh, 2015) future work should leverage our approach to large-scale genetic (Thyme et al., 2019) or pharmacological datasets (Rihel et al., 2010a). Individually, these datasets would provide information on the genetic and molecular basis of behavior across multiple timescales, encompassing processes from sleep to aging. In combination, by identifying mutant and drug-induced phenotypes that cancel each other out (Lamb et al., 2006; Hoffman et al., 2016), these datasets could be used to identify phenotypic suppressors in genetic disease models, an outcome with potential clinical relevance.

## Synthesis

Reviewing Editor: Tatyana Sharpee, The Salk Institute for Biological Studies Decisions are customarily a result of the Reviewing Editor and the peer reviewers coming together and discussing their recommendations until a consensus is reached. When revisions are invited, a fact-based synthesis statement explaining their decision and outlining what is needed to prepare a revision will be listed below. The following reviewer(s) agreed to reveal their identity: David Ehrlich.

The study “Hierarchical Compression Reveals Sub-Second to Day-Long Structure in Larval Zebrafish Behavior” employed a novel approach in zebrafish to assess patterns in swimming behavior across sequences of bouts. The authors categorized swim bouts and pauses into “modules” using cluster analysis, then identified common sequences of modules called “motifs” using hierarchical compression. These modules and motifs varied in frequency across a number of domains (day vs. night, pharmacological agent and dose, genotype). Behavior was categorized based on movement timeseries from 96-well plates, with each fish described as a vector of pixel change counts. These minimalistic data permitted the classification of timeseries into categories of alternating bouts and pauses over millions of cycles. The extraordinary scale of behavior collected was critical for testing the strongest idea in the manuscript, that certain motifs are common across fish and specific to a particular state or dose of drug. The application of hierarchical compression to assess behavioral sequences in zebrafish is novel and of great interest, and the experiments demonstrate the applicability and value of this approach for comparing behavior across conditions. The manuscript was generally well written, the concepts made accessible, and the figures largely clear and informative. The manuscript needs to be improved to address concerns about clustering results and providing statistics on motif frequency.

Major concerns:

The clustering of pauses requires more exploration and support. The authors performed k-means clustering on this scalar data to categorize pauses according to duration - a defensible step for making hierarchical compression practical - resulting in 5 clusters. Regardless, the resulting cutoffs between clusters (0.16, 1.20, 3.72, 20.04 sec) appear arbitrary. The authors claim to “identify” 5 pause modules (L701), but I understand these modules to be imposed on the data. If the resulting cutoffs are effectively arbitrary, the authors should be more up-front about this critical simplification. Given this apparent arbitrariness and that the definition of motifs is sensitive to the categorization of pauses, it is essential to test whether conclusions about motifs are robust to the clustering of pauses. For example, inactive modules are defined collectively for day and night data. Consequentially, inactive modules are more evenly distributed in nighttime data than daytime data (Figure 2D). Daytime pauses are effectively chosen from 3 modules (1-3), while nighttime pauses are chosen from 5. I would think this simple fact makes daytime data more compressible, ignoring any differences in module sequencing. If pause cutoffs are set to give approximately equally populated inactive modules in daytime rather than nighttime, does compressibility flip to be larger at night than day (Fig 3B)? A key step will be to perform hierarchical compression again with 4 or 6 inactive modules to see whether the data on motif specificity in Figs. 5A and 5B obtain, with similar motifs observed under each drug dose.

The authors should more clearly explain the calculation of enrichment/constraint and calculate statistics to facilitate its interpretation. The authors counted motif observations and “Normalized these counts by calculating whether...” (L579), but “whether” implies a binary. How was the normalization performed? Why does Eq. 3 output +/-3.32 when sigma=0 (L388)?Normalization to counts in shuffled data involved in the computation of “enrichment/constraint” is useful to suggest an observed sequence occurs more often than chance in a block given a set of modules. However, many such blocks were analyzed and ∼50,000 motifs comprised the library. Given that so many blocks were analyzed, some motifs would naturally be repeated more than chance. Can the authors randomly generate a sequence of modules of the same length as the analyzed data across all blocks, in order to give a sense for the largest enrichment/constraint scores for motifs repeated by chance? This approach may provide a false positive rate, which would help assess whether observed motifs are observed significantly more than chance (applicable to Figs. 4C, 5A, 5B).

Minor concerns:

Was data independent across larvae if they could see each other swim? Did correlations of behavior across individuals, and specifically the timing of bouts, vary as a function of proximity of wells? Were fish of a given condition/dose distributed across wells for each experiment in a manner that could avoid this confound?

Abstract: “movements and pauses, termed bouts.” The term ‘bouts’ typically describes active swimming events, connoting the repetition of tail beats that comprises each swim event. Referring to pauses as “inactive bouts” throughout is confusing.

The authors should clarify whether larvae were fed prior to or during the experiment, or if they simply survived on yolk.

How many larvae were used for experiments? Approximately how many larvae died or were unresponsive (L99)?

Figure 1B- By my calculation, 100,000 bouts over 28 daytime hours corresponds to ∼1 Hz swim rate. These data are (importantly) consistent with literature and typically presented as rates. Comparing number of bouts across 28 daytime and 20 nighttime hours is misleading.

Figure 2C left - Are some active modules not plotted? This figure appears only red and is difficult to read.

A matter of taste, but readability is hindered by re-using the colormap for active modules (reds) to show melatonin dose, and reusing the colormap for inactive modules (blues) to show day/night and enrichment/constraint (e.g. Fig. 4B).

Figure 3B- An intriguing result! How did the motif dictionary compare for larvae at night and those treated with melatonin? Is the change in compressibility due to melatonin dose driven by module distribution or module sequence?

L558 apostrophe typo

L604- This claim is critical and it needs context. Is it surprising that only a few motifs were needed to classify day vs. night? Certain modules (e.g. inactive module 5) are almost exclusively observed during the night. A comparison to classification based solely on inactive duration would be useful. How many pauses must be observed to classify day/night with the same confidence?

L609 - To distinguish development from adaptation the authors must vary the day they introduce larvae to the plates.

Figure 4D - Can the authors justify the definition of a “startle” pattern with literature or further explain their logic?

L629 - Why would dose-specific motifs evidence low-affinity receptors? The attribution seems arbitrary. Perhaps this point would be better made in the Discussion when interpreting dose-specific data.

Figure 5A- Why is the best motif for a given dose of melatonin not always most prominent in that dose (e.g. 0.01uM motif is most enriched in control fish)?

L644- What is a “behavioral seizure” and which motif corresponds to it?

It is important to get a sense of how common these motifs are. Can the authors plot in Fig. 3C a marginal showing the probability distribution of motifs across all data?

L685- In this paragraph, can the authors speak to the steps to modify the existing protocol to account for higher-dimensional behavioral data?

Fish can translate a large proportion of body length per frame at 25Hz (ignoring interactions with walls of a well). How many pixels does a larva span? The maximal possible delta pixel is important for interpreting bout data. Do some bouts reach this ceiling? Would higher frame rates be prescribed for future applications?

## Author Response

Synthesis of Reviews:

Computational Neuroscience Model Code Accessibility Comments for Author (Required): The code will be provided

Significance Statement Comments for Author (Required): The statement reads well.

Comments on the Visual Abstract for Author (Required):

n/a

Synthesis Statement for Author (Required):

The study “Hierarchical Compression Reveals Sub-Second to Day-Long Structure in Larval Zebrafish Behavior” employed a novel approach in zebrafish to assess patterns in swimming behavior across sequences of bouts. The authors categorized swim bouts and pauses into “modules” using cluster analysis, then identified common sequences of modules called “motifs” using hierarchical compression. These modules and motifs varied in frequency across a number of domains (day vs. night, pharmacological agent

and dose, genotype). Behavior was categorized based on movement timeseries from 96-

well plates, with each fish described as a vector of pixel change counts. These minimalistic data permitted the classification of timeseries into categories of alternating bouts and pauses over millions of cycles. The extraordinary scale of behavior collected was critical for testing the strongest idea in the manuscript, that certain motifs are common across fish and specific to a particular state or dose of drug. The application of hierarchical compression to assess behavioral sequences in zebrafish is novel and of great interest, and the experiments demonstrate the applicability and value of this approach for comparing behavior across conditions. The manuscript was generally well written, the concepts made accessible, and the figures largely clear and informative. The manuscript needs to be improved to address concerns about clustering results and providing statistics on motif frequency.

Dear Reviewers,

Thank you for your interest in our manuscript. We are grateful for the time you have put into the process, evident from your thoughtful comments. Below, we have divided your concerns into individual points and provided a reply to each. Where appropriate we have performed additional data analysis and made changes to the manuscript and figures. We believe these changes address all concerns and have strengthened our manuscript.

Major concerns:

The clustering of pauses requires more exploration and support. The authors performed k-means clustering on this scalar data to categorize pauses according to duration - a defensible step for making hierarchical compression practical - resulting in 5 clusters. Regardless, the resulting cutoffs between clusters (0.16, 1.20, 3.72, 20.04 sec) appear arbitrary. The authors claim to “identify” 5 pause modules (L701), but I understand these modules to be imposed on the data. If the resulting cutoffs are effectively arbitrary, the authors should be more up-front about this critical simplification.

We are glad that the reviewers recognise the importance of clustering the pauses into modules for subsequent analysis and interpretation. We would, however, like to emphasise that our clustering approach is fully unsupervised. Thus, our clusters are identified from, not imposed upon, our data.

As detailed in the methods (line 312) to identify our inactive (and active) modules, we designed an algorithm based upon Fred and Jain, 2002 and 2005, which executes the following steps:

1. Randomly sample a set of probe bouts

2. Over 200 iterations (with n and k varying uniformly):

a. Randomly sample n bouts (from the full set of bouts)

b. Fit a Gaussian mixture model with k components c. Assign probe points to components

d. Accumulate evidence - pairwise occurrence of probe points in the same cluster

3. Apply average link clustering to the evidence accumulation matrix (probe points x probe points)

4. Determine the final number of clusters by using the maximum differentiated linkage distance to cut the resultant dendrogram

This approach is appropriate for our large datasets (30,900,018 active and 30,900,418 inactive bouts), as it allows us to implicitly explore the effects of and interaction between the bouts sampled, the number of bouts sampled and the number of mixture

components, and ultimately combine this information across 200 models to an aggregate solution.

Ultimately, we do, however, recognise that clustering is an inherently challenging problem and hope that our discussion of alternative approaches (Line 770 onwards) and openness of data and code sharing through online repositories will stimulate further work in this area.

Given this apparent arbitrariness and that the definition of motifs is sensitive to the categorization of pauses, it is essential to test whether conclusions about motifs are robust to the clustering of pauses. For example, inactive modules are defined collectively for day and night data. Consequentially, inactive modules are more evenly distributed in nighttime data than daytime data (Figure 2D). Daytime pauses are effectively chosen from 3 modules (1-3), while nighttime pauses are chosen from 5. I would think this simple fact makes daytime data more compressible, ignoring any differences in module sequencing.

The reviewers are correct to recognize that a difference in compressibility could arise from differences in either module or motif usage.

Indeed, to determine the contribution of each we calculate “Δ compressibility” (the difference in compressibility between each animal’s real and shuffled data). From this metric we demonstrate that the difference between the day and the night is, as the reviewer suggests, predominantly due to differences in module usage (Lines 574).

To make this clearer in the manuscript we have changed the word “selection” in Line 576, to “distribution", and added a concise summary of our compressibility and Δ compressibility findings (Line 593).

If pause cutoffs are set to give approximately equally populated inactive modules in daytime rather than nighttime, does compressibility flip to be larger at night than day (Fig 3B)?

We understand the reviewers concern regarding how our bout classification impacts upon our compression results. Hopefully, as detailed above, we have now sufficiently emphasised the unsupervised nature of our bout classification and made clearer how Δ compressibility addresses differences in module distribution between contexts.

Here, we understand the reviewers to be asking “is day or night behaviour more compressible, module differences aside?”. This question is addressed by a comparison of Δ compressibility, which demonstrates that even once differences in module distribution are accounted for, the daytime data is more compressive and hence more sequence rich (Supplemental Figure 5e).

A key step will be to perform hierarchical compression again with 4 or 6 inactive modules to see whether the data on motif specificity in Figs. 5A and 5B obtain, with similar motifs observed under each drug dose.

Although we believe an unsupervised module classification approach to be appropriate for our aim, the reviewers raise an interesting point; how would the most salient motifs for a comparison compare when drawn from a motif library built from different modules?

Here, we are happy to provide data from a preliminary version of our analysis. This dataset included data from a second hypocretin mutant, which we later excluded due to poor quality of tracking. Aside from the additional data, we would like the reviewers to note that there are many differences between this and our final analysis including different thresholding parameters and the use of an alternative, less suitable, clustering method.

In this model, we identified 10 active and 6 inactive modules. To test how motif usage would differ between this and our final module set (5 active and 5 inactive), we compared the most salient control vs PTZ motif from each set.

The preliminary motif is 3 modules long: active module 7, inactive module 1, active module 7, and thus represents a pair of high amplitude movements broken by the shortest pause.

The usage of this motif (y-axis) is shown below (Reviewer Figure 1) across control and PTZ dosed axis). Each animal is represented by two dots (day and night) with a black cross denoting

each group’s mean.

This result is quite similar to our final analysis which also identifies a seizure motif 3 modules long: active module 2, inactive module 1, active module 4 - again a pair of active bouts broken by the shortest pause. Similarly, the usage of this motif is constrained in control fish and enriched in PTZ dosed fish (Figure 5B, middle panel - a cropped and rotated version of which is shown below to facilitate comparison to the preliminary model).

Overall, this demonstrates that motif libraries built from different module sets can still lead to similar salient motifs. We tentatively suggest that this example

demonstrates our analysis is likely robust to changes in our unsupervised module classification.

The authors should more clearly explain the calculation of enrichment/constraint and

calculate statistics to facilitate its interpretation. The authors counted motif observations and “Normalized these counts by calculating whether...” (L579), but “whether” implies a binary. How was the normalization performed?

We thank the reviewers for highlighting the need for a better explanation of our enrichment/constraint metric.

We have amended the main text to make its calculation clearer (lines 617) and removed

the word “whether” as suggested:

"To test this hypothesis, we counted the number of times each larva or set of paired shuffled data used each motif within each time frame (e.g. day or night) and then normalised these counts by calculating the deviation of the real from shuffled counts, as well as the deviation of each shuffle from the other shuffles, a metric we termed enrichment/constraint.”

In the methods we have also included a sample calculation to aid interpretation (line

389):

"For example, for a given animal and time window, if a motif was used 10 times in the real data and a mean of 4.5 times in the 10 sets of paired shuffled data (standard deviation 1.58), enrichment/constraint would equal 3.48.”

10 − 4.5

3.48 =

1.58

Why does Eq. 3 output +/-3.32 when sigma=0 (L388)?

When there is no standard deviation in the shuffled counts (sigma = 0), Eq.3 (the enrichment/constraint calculation) will output either positive or negative infinity. To facilitate subsequent working, infinite values were replaced with a constant value of √(1

+ number of shuffled counts) = √(11) = {plus minus} 3.32.

We acknowledge that this is a simple replacement scheme; however, in our real data infinite values constituted only 2.2% of all enrichment/ constraint scores.

We have adjusted line 396 to better explain this:

"When comparing the shuffled data to itself, each shuffle (now x_i) was excluded from s _i and σ_si. Infinite values occurred when there was no standard deviation in the σ_si counts and thus σ_si equalled zero. To facilitate subsequent working, infinite values were replaced with a constant value of √(1 + number of shuffled counts) = √(11) = {plus minus} 3.32. Note that in the real data, infinite values constituted only 2.2% of all enrichment/ constraint scores.”

Normalization to counts in shuffled data involved in the computation of “enrichment/constraint” is useful to suggest an observed sequence occurs more often than chance in a block given a set of modules. However, many such blocks were analyzed and ∼50,000 motifs comprised the library. Given that so many blocks were analyzed, some motifs would naturally be repeated more than chance. Can the authors randomly generate a sequence of modules of the same length as the analyzed data

across all blocks, in order to give a sense for the largest enrichment/constraint scores for motifs repeated by chance? This approach may provide a false positive rate, which would help assess whether observed motifs are observed significantly more than chance (applicable to Figs. 4C, 5A, 5B).

We are glad that the reviewers find our enrichment/constraint metric useful and agree that across such a large dataset it is likely that some motifs will be repeated more or less than chance and thus have non-zero enrichment/constraint scores.

To determine the distribution of chance enrichment/constraint scores, we shuffled every animals’ entire dataset 10 times, and then calculated enrichment/constraint as for the real data.

The results of this process are shown in Figure 4a where probability density functions show the probability of observing motifs at different enrichment/constraint scores.

As seen in the figure, the real and shuffled enrichment/constraint scores diverge before

{plus minus} 4. This demonstrates that in the shuffled data values above / below {plus minus} 4 are seen far less commonly than in the real data. To quantify this difference, we compared the kurtosis of each animal’s real and shuffled distribution (Figure 4a, insert), demonstrating that the kurtosis of the real data is significantly higher.

Minor concerns:

Was data independent across larvae if they could see each other swim? Did correlations of behavior across individuals, and specifically the timing of bouts, vary as a function of proximity of wells?

We thank the reviewer for raising this issue. To address the reviewer’s comment, we:

• Took a sample of 24 wild-type fish from our data

• Computed the pairwise correlation between their active bout starts across 24hrs

• Defined each pairwise comparison as either an “autocorrelation", a

"neighbour” (i.e. in an adjacent well of the 96 well plate, diagonals excluded) or

"distant” (i.e. in non-adjacent wells)

• Plotted the mean and standard deviation for each group

• Note that the y-axis is cropped from 1, where autocorrelation peaks, to 0.1.

As can be seen in the resultant figure (reproduced below) there is no significant effect of well proximity upon the timing of active bout starts. This result is broadly consistent with

results from Dreosti et al., 2015, who found that at this age larvae show no avoidance or

preference towards other larvae freely swimming behind a glass window.

We agree with the reviewer that this is an important consideration and thus have added the following text to the manuscript (line 463), as well as this figure to the supplement (Supplementary Figure 1b).

"We found no correlation of active bout initiation between larvae in adjacent wells of the

96-well plate (Supplementary Figure 1b), suggesting no interactions among larvae.”

Were fish of a given condition/dose distributed across wells for each experiment in a manner that could avoid this confound?

For the hypocretin experiments larvae were plated randomly as we were blind to genotype at this point. For the pharmacological experiments, we dosed each row of the plate with a successively higher dose. To make this clearer we have included the following line in the methods (line 93):

"Following two control rows, each row was dosed with a successively higher concentration

of test compound.”

As shown above, there is no significant effect of well proximity upon the timing of active bout starts.

Abstract: “movements and pauses, termed bouts.” The term ‘bouts’ typically describes active swimming events, connoting the repetition of tail beats that comprises each swim event. Referring to pauses as “inactive bouts” throughout is confusing.

Whilst we concede that in the zebrafish literature the term bout does typically describe a swimming movement, in the sleep field, which we hope will take interest in our paper, it is common to refer to bouts of inactivity (for example rest or sleep bouts). We therefore prefer to keep this terminology.

The authors should clarify whether larvae were fed prior to or during the experiment, or if they simply survived on yolk.

We thank the reviewers for noting our omission, larvae were not fed either prior to or during experiments. We have included this information in the text (line 95):

"Larvae were not fed either prior to or during experiments.”

How many larvae were used for experiments? Approximately how many larvae died or were unresponsive (L99)?

We thank the reviewers for suggesting we include this information. We have added the following to the methods section (line 102).

"In the wild-type experiments we additionally excluded wells in which a bubble had formed under the plastic lid. The number of larvae excluded / used for each set of experiments is as follows: wild-type -- 164/288 (all exclusions were due to bubble artefacts), hypocretin -

0/180, melatonin - 0/96, PTZ - 44/96 (doses of 10, 15 and 30mM were lethal to all tested

larvae).”

Figure 1B- By my calculation, 100,000 bouts over 28 daytime hours corresponds to ∼1 Hz swim rate. These data are (importantly) consistent with literature and typically presented as rates. Comparing number of bouts across 28 daytime and 20 nighttime hours is misleading.

We would like to thank the reviewers for noting that our data are consistent with the broader literature, especially as compared to larvae in larger arenas. We have altered Figure 1b to report Bout Frequency (Hz) as suggested and have highlighted the consistency of our result with the literature in the main text (line 468):

"During the day, wild-type larvae initiated bouts at a mean frequency of 0.89 Hz (Figure 1b) a rate consistent with other assays (Kim et al., 2017), and tended to use short, sub-second long inactive bouts (Figure 1c). During the night, larvae displayed a mean bout frequency of only 0.07 Hz (Figure 1b) and utilised longer inactive bouts, on the order of seconds to minutes (Figure 1c).”

Figure 2C left - Are some active modules not plotted? This figure appears only red and is difficult to read.

We are grateful to the reviewers for spotting this subtle error. We have corrected figure

2C to include all active modules.

The reviewer is right to point out that there is a lot of data displayed here (each matrix is

124 x 4,319,953). It is, however, our intention in this panel to show the data in as unprocessed a form as possible to illustrate our starting point for subsequent analysis. Furthermore, showing the data in this way allows readers to assess the consistency of later findings across individual animals. We hope that the two subsequent panels (Figure

2d and e), aid in the interpretation of this figure.

A matter of taste, but readability is hindered by re-using the colormap for active modules (reds) to show melatonin dose, and reusing the colormap for inactive modules (blues) to show day/night and enrichment/constraint (e.g. Fig. 4B).

Unfortunately, the use of a single colormap was decided upon early in the project.

As kindly noted in the synthesis statement, our figures are “largely clear and informative”. We hope that the reviewers appreciate our efforts towards this, including clear figure legends, consistency across panels and an effort to include only key findings in the main figures.

Figure 3B- An intriguing result! How did the motif dictionary compare for larvae at night and those treated with melatonin?

The reviewers raise an interesting question, specifically do melatonin treated larvae use similar behavioural motifs to larvae at night?

To address this question, we:

1. Calculated the mean enrichment/constraint for each motif across wild-type larvae during the night (a 1 x 46,554 vector)

2. Calculated the Euclidean distance between each wild-type sample (separately day and night) and this mean

3. Calculated the Euclidean distance between each melatonin sample (during the day) and this mean

The figure below shows these distances:

As expected, wild-type animals are further from the night mean during the day and closer during the night. Interestingly, with increasing melatonin dose motif usage shifts closer to the WT night mean, though remains distinguishable. This result suggests that melatonin induces night-like motif usage.

Note: we believe the difference between the wild-type day and melatonin control fish to be a result of the 0.2% DMSO applied to the melatonin control fish. As our melatonin is dissolved in DMSO, this is the most appropriate control for this experiment.

While we believe this finding to be of interest, we have decided not to include it in the manuscript, as we are not confident that this is the best way to compare motif usage similarity. In future, we hope to find a better approach which would allow us to compare motif similarity across larger datasets of drugs and mutants.

Is the change in compressibility due to melatonin dose driven by module distribution or module sequence?

We thank the reviewers for noting our omission of this result from the text. To rectify this, we have added the following line to the results section (line 582):

"We found that melatonin decreased day compressibility to night-time levels (Figure 3b), a difference primarily due to shifts in module distribution (Supplemental Figure 5e).”

As noted in response to a comment earlier we have also added a concise summary of our compressibility and Δ compressibility findings (line 593), which we hope clarifies our results.

L558 apostrophe typo

We thank the reviewers for noticing this error, which we have now corrected.

L604- This claim is critical and it needs context. Is it surprising that only a few motifs were needed to classify day vs. night? Certain modules (e.g. inactive module 5) are almost exclusively observed during the night. A comparison to classification based solely on inactive duration would be useful. How many pauses must be observed to classify day/night with the same confidence?

Though we were also impressed by this result, we believe that it is well contextualised in the paper.

To reiterate, we show that using motif enrichment/constraint we can distinguish day/night behaviour with only 0.2% error with 15 motifs. We contextualise this result by comparison to both a majority class classifier (50% error) and random 15-motif subsets (9.25% error).

Furthermore, we demonstrate that though a module-based classifier performs well (1.61% error), it is still inferior to a motif-based classifier; a generalisable finding across our datasets (Figure 5c).

Following the reviewers request for a “classification based solely on inactive duration” we additionally built a module classifier using only inactive module usage. This achieved a

2.42% classification error, poorer than either the overall (active and inactive) module-

based or motif classifiers.

As our original module-based classifier achieved its best performance using all 10 modules (5 active and 5 inactive) (Table 3), we have decided not to include the data from this purely inactive-module based model, which achieves worse classification and does therefore not add substantially to our manuscript.

L609 - To distinguish development from adaptation the authors must vary the day they introduce larvae to the plates.

The reviewers make a valid point. We have amended the text where appropriate. For example (line 650):

"Next, we examined how motif usage changed over time by comparing consecutive days and nights (5-6dpf).”

Figure 4D - Can the authors justify the definition of a “startle” pattern with literature or further explain their logic?

To address the reviewer’s comment we have added the following (line 662):

"We interpret these motifs as startle-like as they consist of long pauses interrupted by high- amplitude movements and primarily occur at the lighting transitions (Figure 4d), stimuli known to elicit startle behaviours in larval zebrafish (Burgess and Granato, 2007; Emran et al., 2010).”

L629 - Why would dose-specific motifs evidence low-affinity receptors? The attribution seems arbitrary. Perhaps this point would be better made in the Discussion when interpreting dose-specific data.

At the reviewer’s suggestion we have removed this from the text.

Figure 5A- Why is the best motif for a given dose of melatonin not always most prominent in that dose (e.g. 0.01uM motif is most enriched in control fish)?

While the reviewer is correct to note that motif usage (enrichment) is an important factor for distinguishing between doses, decreased motif usage (constraint) can be just as relevant. For example, the best 5mM PTZ motif (Figure 5b) is comparatively enriched at both 2.5 and 7.5 mM doses.

This specific case, however, is due to our approach for selecting the best motifs between test conditions which we have made clearer in the methods (line 404):

"For each comparison we defined the first motif chosen by mRMR as the best motif. To determine the best motifs among test conditions we excluded control data. For example, to determine the best 0.01μM melatonin motif, we compared this group’s data to all larvae dosed with melatonin at other doses (Figure 5a). For completeness, we still plot the control enrichment/constraint scores for these motifs.”

L644- What is a “behavioral seizure” and which motif corresponds to it?

We define the motifs shown in Figure 5b (control, 2.5 and 5mM best motifs) as behavioural seizures as they are constrained in control larvae (used less than in paired shuffled data) and enriched in PTZ dosed larvae.

Prior work in larval zebrafish has demonstrated that PTZ does induce “ictal and interictal- like electrographic discharges” as determined by extracellular recordings (Baraban et al.,

2005).

Here, however, we define these motifs as “behavioural seizures” as we don’t show any

evidence directly linking these motifs to epileptiform neuronal activity.

It is important to get a sense of how common these motifs are. Can the authors plot in

Fig. 3C a marginal showing the probability distribution of motifs across all data?

Following the reviewers request we have added a new supplementary panel (Supplementary Figure 5d), showing the probability distribution of motif usage as a function of motif length across all real data. For comparison, we have additionally plotted the results from our shuffled datasets.

Comparison of the real to shuffled data yielded an interesting finding, which we have now included in the manuscript (line 607):

"In the real data, longer motifs tended to be used more frequently than in the shuffled data (Supplementary Figure 5d), demonstrating the likely behavioural relevance of these sequences.”

L685- In this paragraph, can the authors speak to the steps to modify the existing protocol to account for higher-dimensional behavioral data?

As suggested, we have included a sentence to make this clearer (line 751):

"Once such high dimensional data had been assigned to modules, our compression and motif enrichment/constraint approach could be applied in the same manner as here.”

Fish can translate a large proportion of body length per frame at 25Hz (ignoring interactions with walls of a well). How many pixels does a larva span? The maximal possible delta pixel is important for interpreting bout data. Do some bouts reach this ceiling?

The largest delta pixel value we observe is 165. As shown by the distribution of active bout maximum values (Supplementary Figure 2a), values so high are extremely rare. Indeed only 1 value of 165 is recorded across our entire dataset of 30,900,018 active bouts.

As shown by the distribution of active bout mean values (Supplementary Figure 2a) most delta pixel changes are between 3 (minimum observed value) and 30.

To make this clearer we have included the following line in the methods (line 222):

"Following this step, the maximum Δ pixels value observed was 165. Most Δ pixels values

were between 3 (minimum observed value) and 30 (Supplementary Figure 2a).”

Would higher frame rates be prescribed for future applications?

As mentioned in the discussion, more detailed behavioural measures would likely yield additional insights (line 743).

However, an alternative approach (line 776), would be to generate a mapping between our high-throughput and other higher-resolution assays (for example Marques et al.,

2018). Thus, allowing users to use “data from our set-up to prioritise pharmacological

compounds or mutants for postural analysis”.
