# Supplementary figures and images for: Hierarchical Compression Reveals Sub-Second to Day-Long Structure in Larval Zebrafish Behavior
Source: eNeuro. 2020 Jul 13;7(4):ENEURO.0408-19.2020. doi: 10.1523/ENEURO.0408-19.2020 (PMC7405074; doi:10.1523/ENEURO.0408-19.2020)

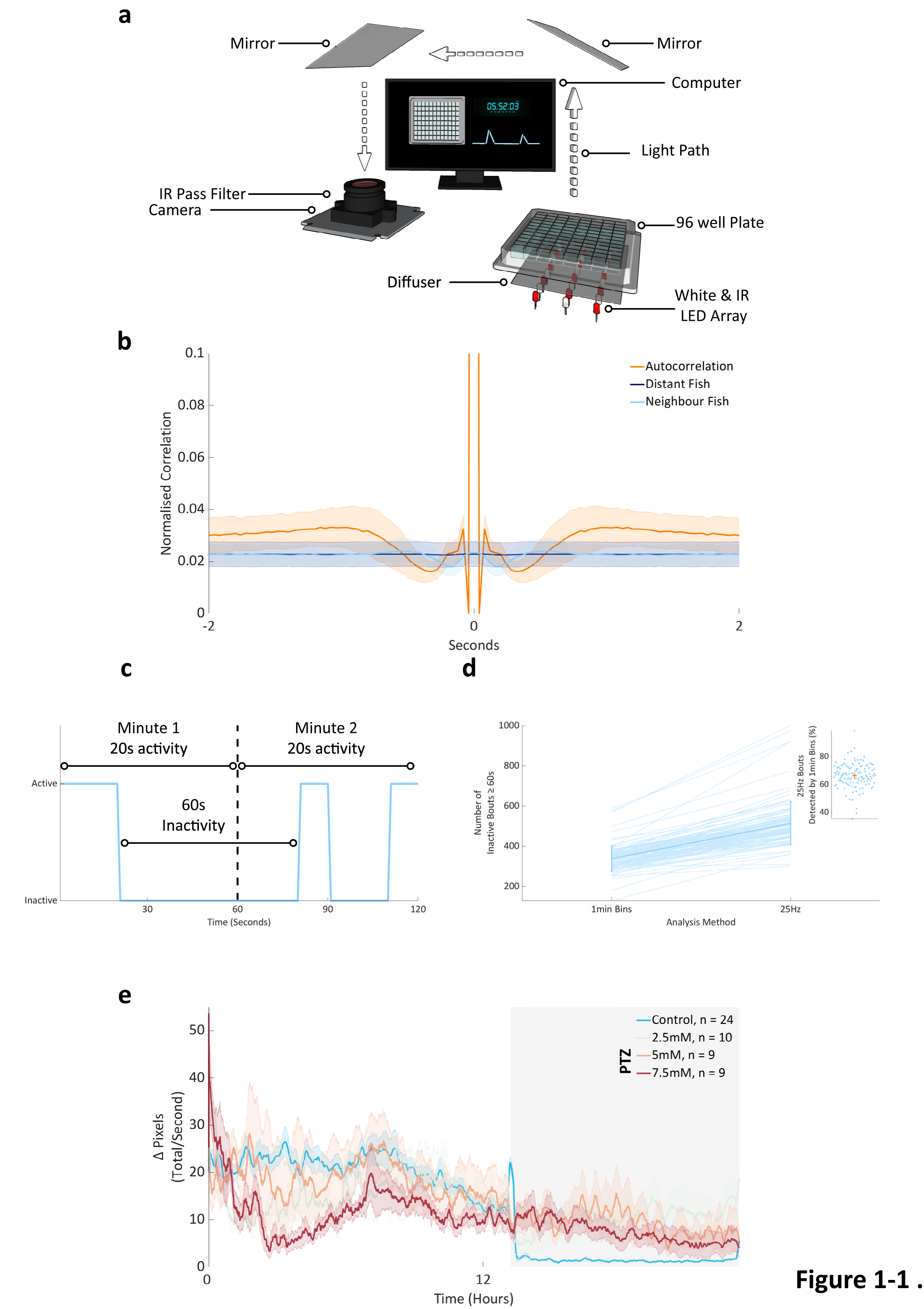

Supplement: Extended Data Figure 1-1 — Behavioral set-up and analysis. A, Schematic of our behavioral set-up. Note that aside from the computer, the set-up is fully enclosed. Not shown to scale. IR, infrared; LED, light emitting diode. B, Normalized temporal correlation of active bout starts between 24 wild-type larva (6 dpf) across 24 h. Pairwise correlations were computed and then grouped into three groups: autocorrelation (orange); neighbor fish, defined as larva in adjacent wells, diagonals excluded (light blue); and distant, non-neighbor fish (dark blue). Data from each group are plotted as a mean (bold line) and SD (shaded surround). Note that the y-axis is cropped from 1, where autocorrelation peaks, to 0.1. C, A fictive illustration of zebrafish behavior (blue line). Two minutes of data are shown divided by a black dashed vertical line. A 1-min binning approach would score both minutes as 20 s of activity and miss the 60-s period of inactivity in between. This latter loss leads to a discrepancy in the number of periods ≥60 s between the 1-min bin and 25-Hz methods (see D). D, The number of inactive periods ≥60 s for each of 124 wild-type animals is shown, as determined by both a 1-min bin and 25-Hz approach. Data are from each animal’s entire recording period (4–7 dpf). Data for each animal is shown as a pale blue line overlaid with a bold line showing the population mean and SD. Inset, The percentage of the 25-Hz counts detected by the 1-min bin method per animal. Each animal’s data are shown by a circle. An orange cross marks the population mean. E, Average activity across one day (white background) and night (dark background) for larvae exposed to either H2O (control) or a range of PTZ doses immediately prior to tracking at 6 dpf. Data for each larva was summed into seconds and then smoothed with a 15-min running average. Shown is a mean summed and smoothed trace (bold line) and SEM (shaded surround); n denotes the number of animals per condition. Download Figure 1-1, TIF file. [file enu-eN-NWR-0408-19-s01.tif]

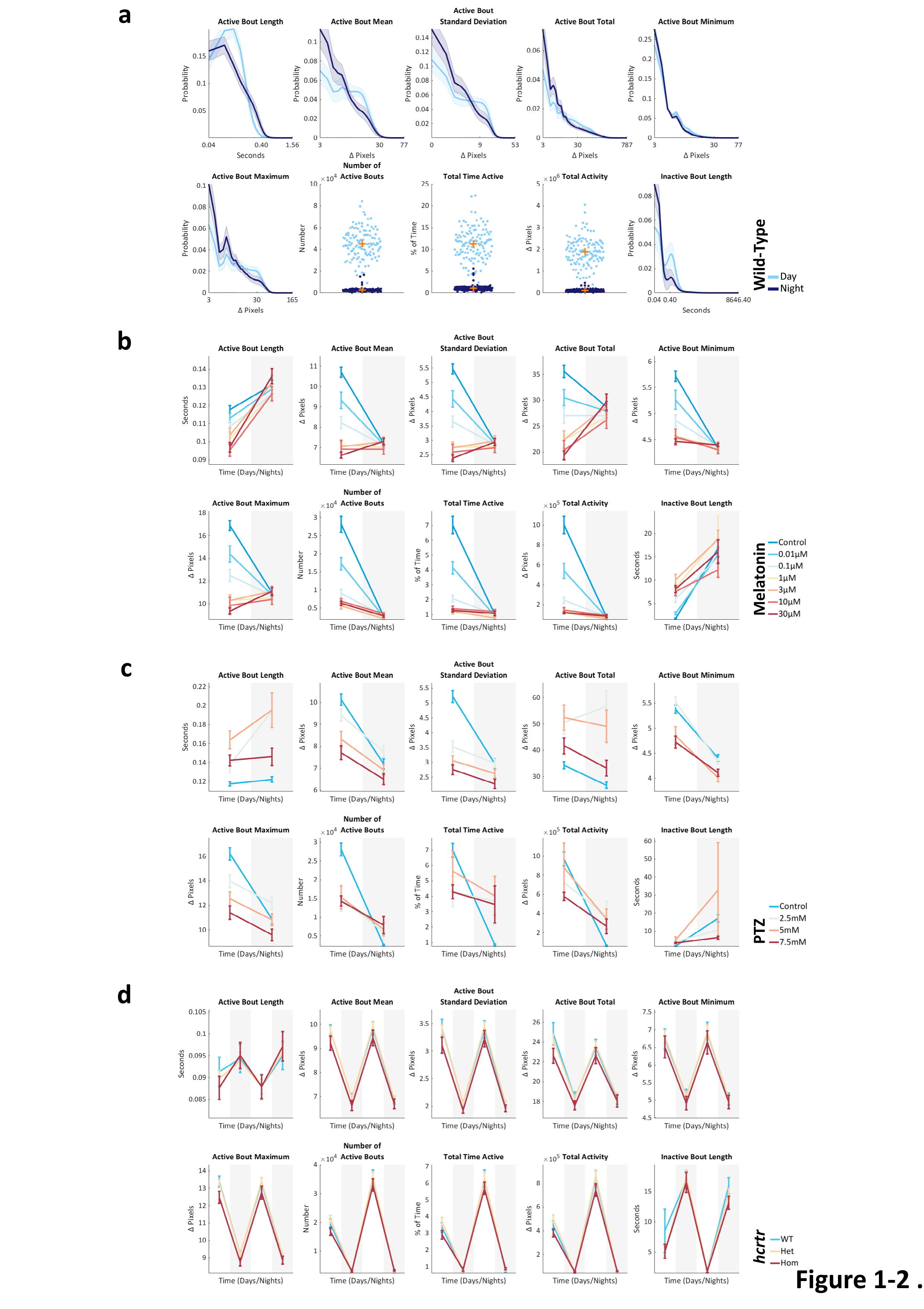

Supplement: Extended Data Figure 1-2 — Bout Features. A, Bout feature distributions during the day (light blue) and the night (dark blue). For the probability curves, each animal’s data were fit with a pdf. Shown is a mean pdf (bold line) and SD (shaded surround) with a log scale on the x-axis. For the scatter plots, each larva’s mean value across the days or nights (5–6 dpf) is shown as a light blue (day) or dark blue circle (night). An orange cross marks each population’s mean. Of the pdfs, only the mean day and night active bout total and inactive bout length pdfs were consistently significantly different across three independent experiments (p < 0.01; two-sample Kolmogorov–Smirnov test); n = 124 wild-type larvae. B, Melatonin bout feature means. A mean was taken per animal per feature, and day or night (6 dpf). Shown is a population mean and SEM during the day (white background) and the night (grey background). Control, DMSO; n = 24 controls then n = 12 per dose. C, PTZ bout feature means, as in B. Control, H2O; n = 24 controls then n = 10 (2.5 mM), n = 9 (5 mM), and n = 9 (7.5 mM). D, hcrtr bout feature means as in B, for days (white background) and nights (grey background) 5–6 post fertilization. hcrtr-/- mutants had significantly lower mean values compared to both hcrtr+/+ and hcrtr-/+ for the following active bout features: length, SD and total (p < 0.05 for all comparisons, Dunn–Sidak corrected four-way ANOVA, adjusted for the following factors: day/night, development, and experimental repeat). No features differed significantly between hcrtr-/+ and hcrtr+/+; n = 39, 102, and 39; for WT, hcrtr+/+; Het, hcrtr-/+; and Hom, hcrtr-/-, respectively. Download Figure 1-2, TIF file. [file enu-eN-NWR-0408-19-s02.tif]

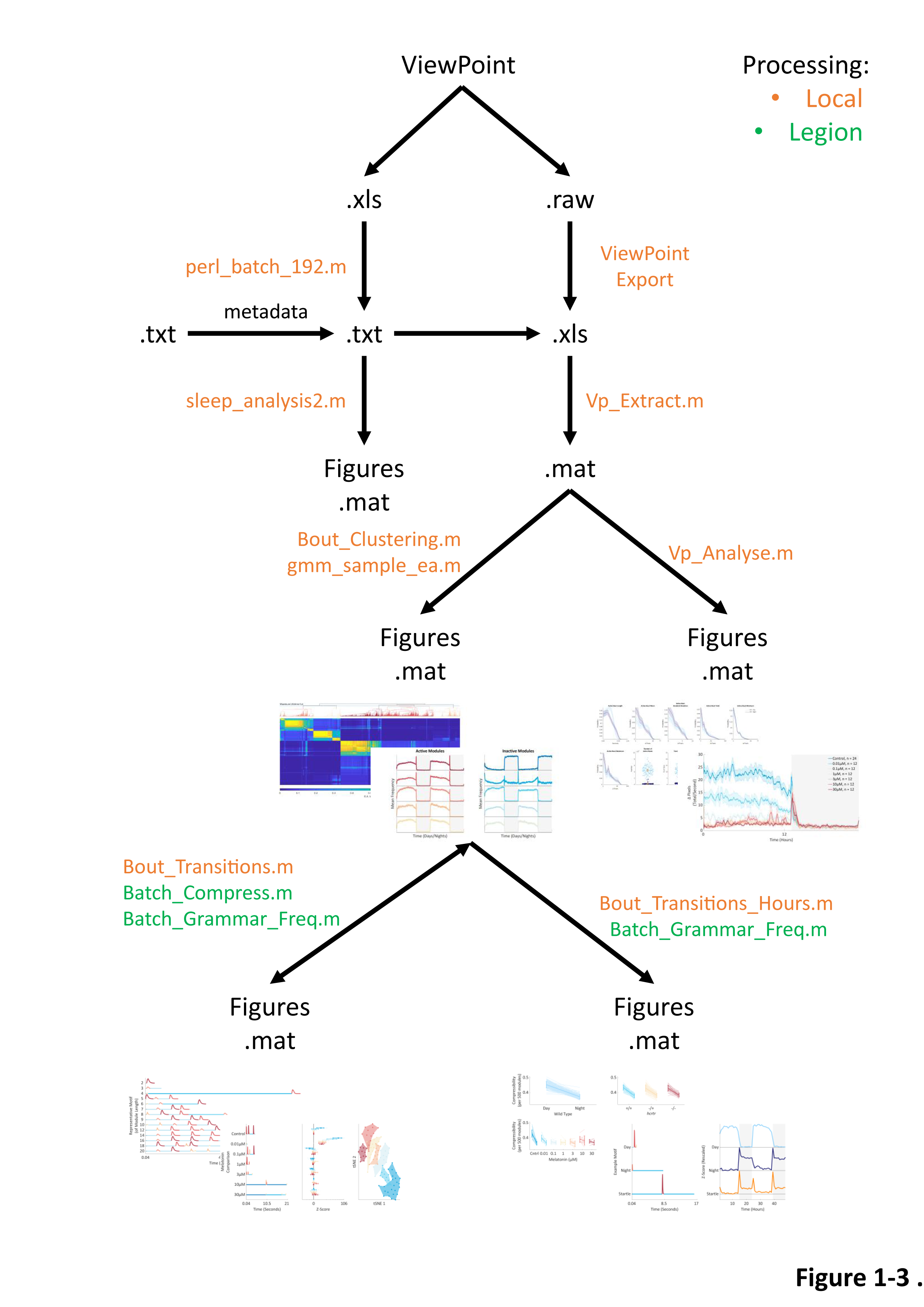

Supplement: Extended Data Figure 1-3 — Analysis framework. Flow diagram depicting the steps of our analysis framework. Data are output from our behavioral set-up (ViewPoint) in the form of a .xls file. perl_batch_192.m organizes these data to a .txt format. Experiment metadata (e.g., animal genotypes) are supplied in the form of a .txt file. The 1-min bin method uses sleep_analysis2.m to produce figures and statistics from these two .txt files. The 25-Hz method exports .raw data from ViewPoint to produce .xls files. Vp_Extract.m reorganizes these, using .txt data, to a .mat file which can be input to either Vp_Analyse.m or Bout_Clustering.m. Vp_Analyse.m produces figures and statistics. Bout_Clustering.m uses the clustering function gmm_sample_ea.m to assign data to modules, produce figures, and calculate statistics, Bout_Clustering.m’s output can be input to Bout_Transitions.m, which compresses full modular sequences by calling Batch_Compress.m and Batch_Grammar_Freq.m. The motifs identified from this approach can be input to Batch_Transitions_Hours.m which compresses 500 module chunks and uses Batch_Grammar_Freq.m to count motif occurrences per hour. With the exception of the 1-min bin method (sleep_analysis2.m), two example figures are shown for each figure producing step. All code can be run locally, although for speed several steps (indicated in green) are best run on a cluster computer. Download Figure 1-3, TIF file. [file enu-eN-NWR-0408-19-s03.tif]

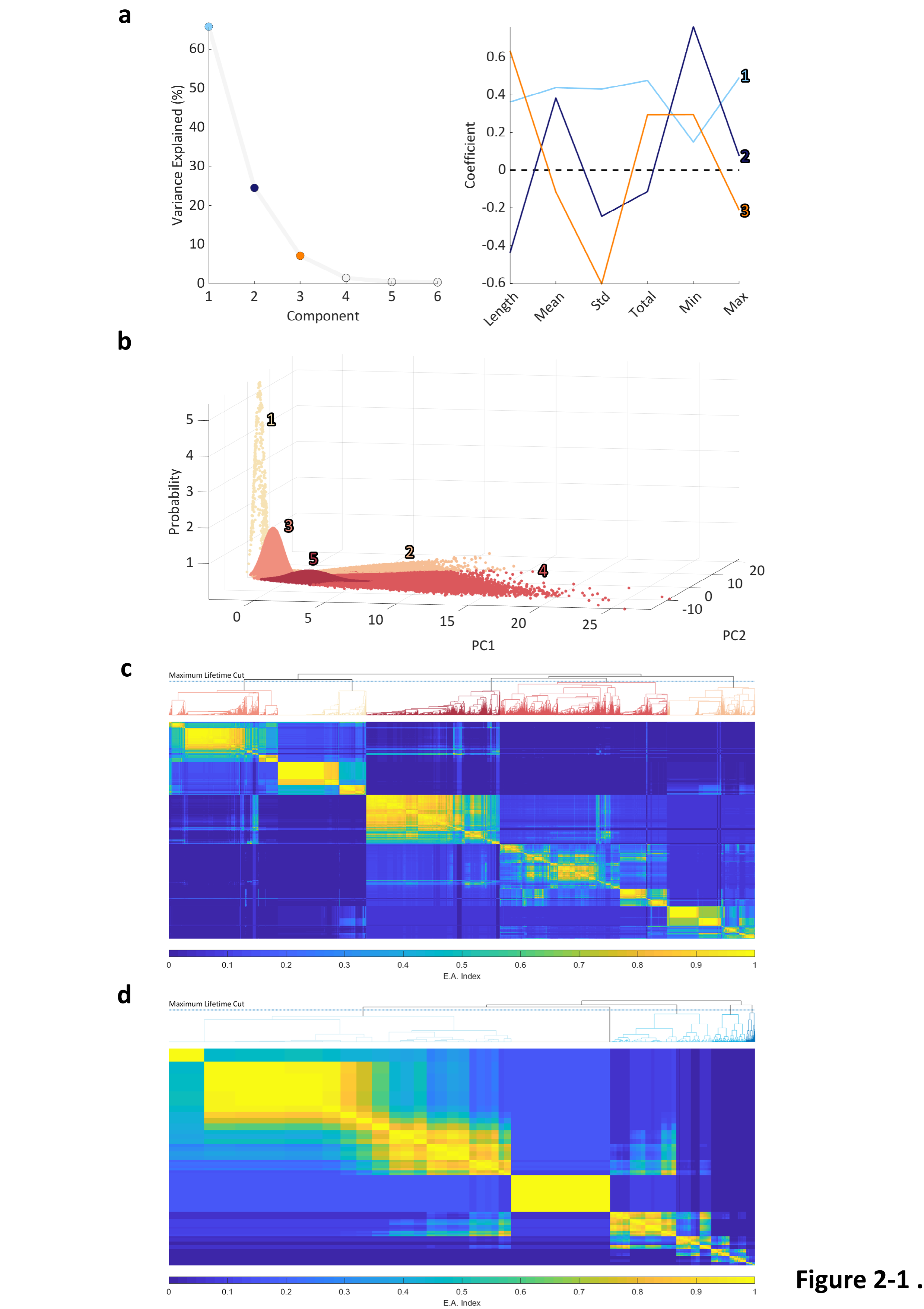

Supplement: Extended Data Figure 2-1 — Evidence accumulation-based clustering. A, left, Scree plot showing the percentage of variance explained by each principal component from the active bout data. The first three principal components, the knee point of the curve, were kept for subsequent analysis. The colors of these points refer to the right panel. Right, Each of the three retained component’s coefficients for the different active bout parameters is shown. B, The active bouts within each module were fit by Gaussian distributions. Each active bout is shown in a 3D space of PC1, PC2, and probability. Each bout is numbered and colored by its module assignment. C, Evidence accumulation (E.A.) matrix for the 40,000 active probe points (matrix dimensions are thus 40,000 by 40,000). A higher E.A. index indicates a higher frequency of pairwise occurrences in the same cluster across 200 Gaussian mixture models. This matrix was clustered hierarchically, and a maximum lifetime cut was made to determine the final number of modules. The dendrogram above shows all 40,000 leaves and is colored by mean module length from shortest (lightest) to longest (darkest) as in other figures. 500. Evidence accumulation matrix for the inactive bouts. Download Figure 2-1, TIF file. [file enu-eN-NWR-0408-19-s04.tif]

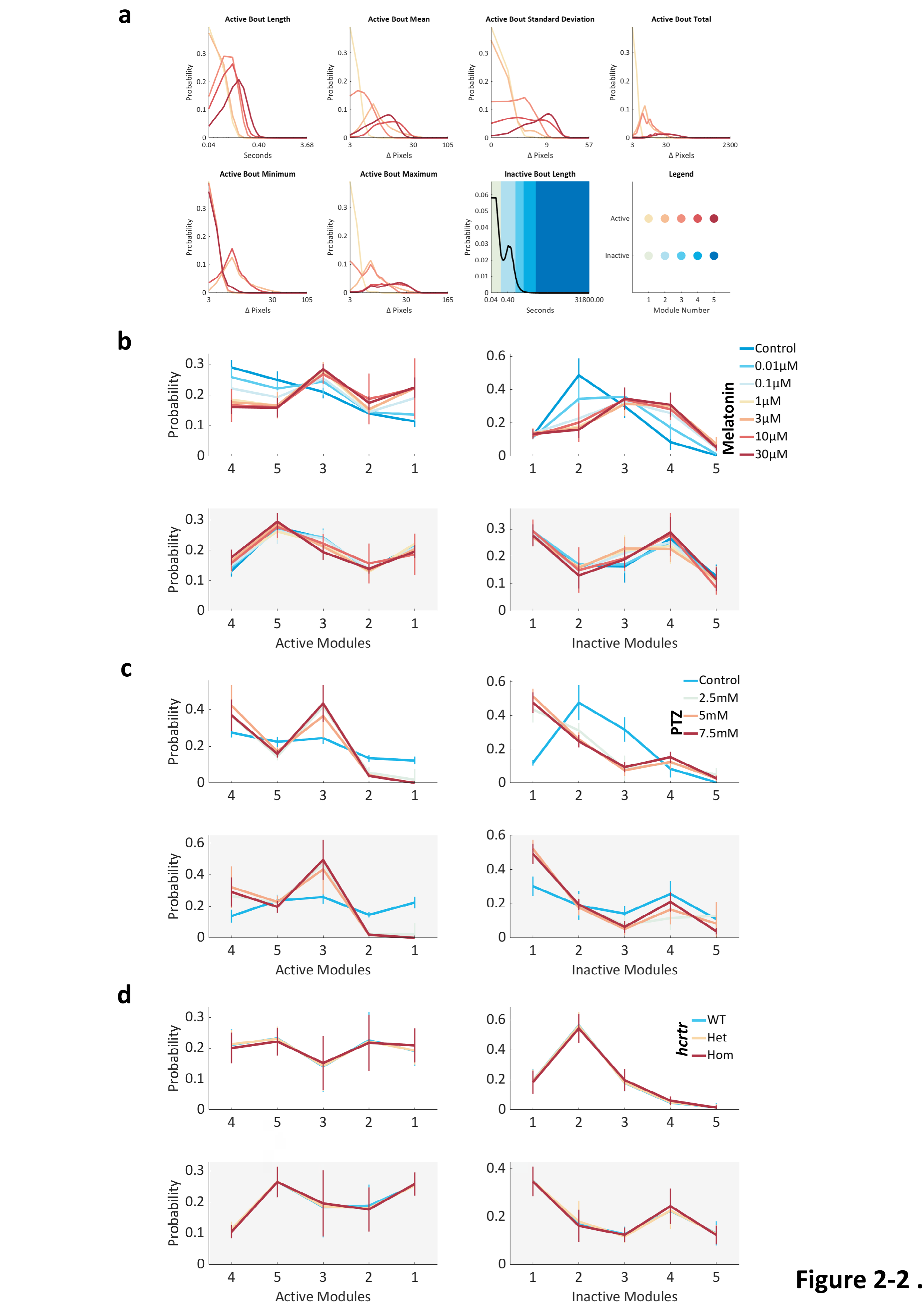

Supplement: Extended Data Figure 2-2 — Behavioral modules. A, pdfs for each bout feature by module. All features are shown on a log x-axis. The legend panel indicates each module’s color. B, Melatonin module probabilities during 6 dpf day (upper panels) and night (lower panels) for both the active (left) and inactive (right) modules. Shown is a mean and SEM for each group, colored according to the legend. Active modules are sorted from highest to lowest by average wild-type day probability, based upon wild-type data in Figure 2D. Inactive modules are sorted by increasing mean length. Control, DMSO; n = 24 controls then n = 12 per dose. C, PTZ data as in B, with H2O (control); n = 24 controls then n = 10 (2.5 mM), n = 9 (5 mM), and n = 9 (7.5 mM). D, hcrtr data as in B, with mean values across 5 and 6 dpf. No module probabilities differed significantly among genotypes (full four-way ANOVA, with the following factors: genotype, day/night, development, and experimental repeat); n = 39, 102, and 39; for WT, hcrtr+/+; Het, hcrtr-/+; and Hom, hcrtr-/-, respectively. Download Figure 2-2, TIF file. [file enu-eN-NWR-0408-19-s05.tif]

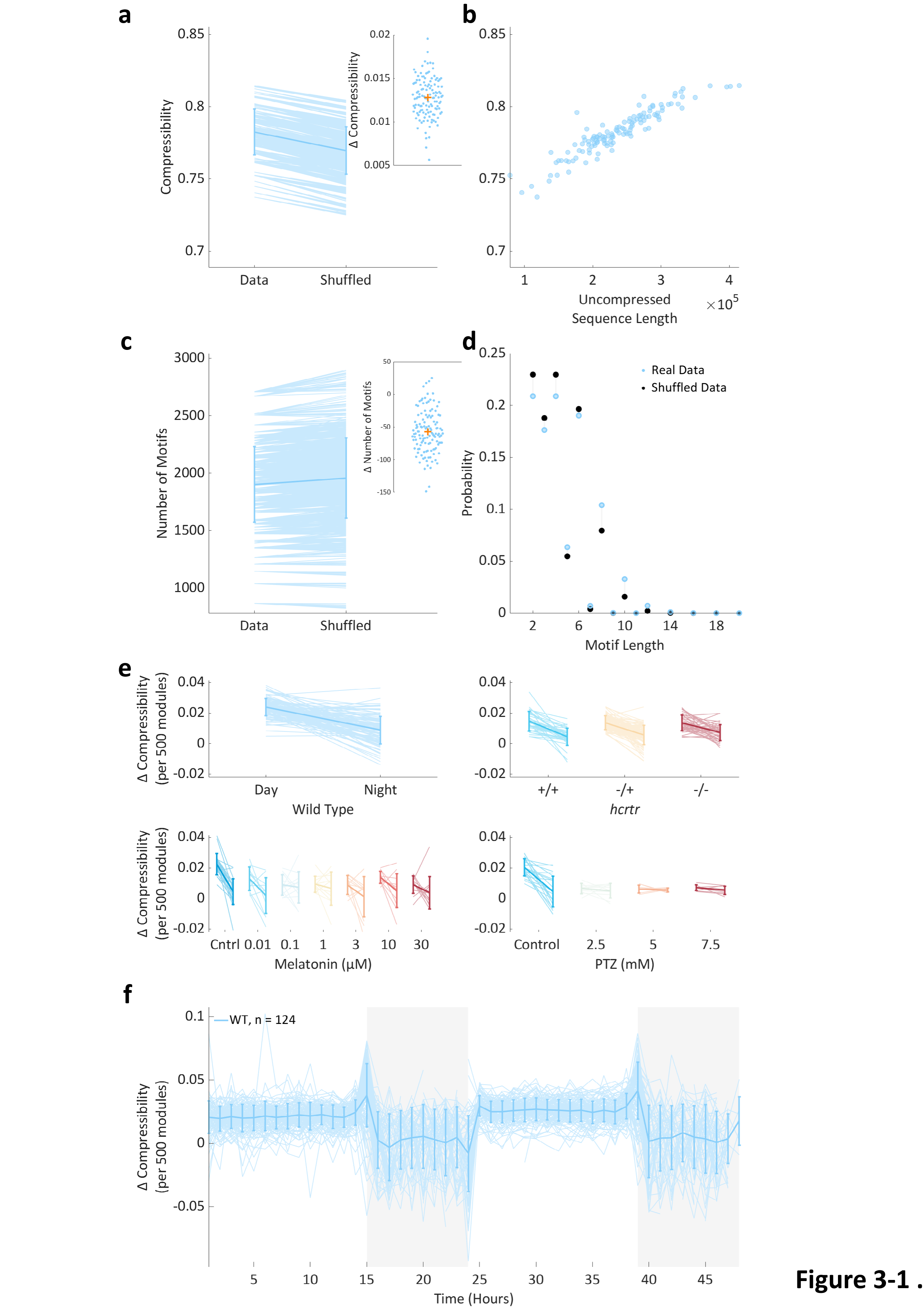

Supplement: Extended Data Figure 3-1 — Hierarchical compression metrics. A, The compressibility (y-axis) of the real wild-type data is higher than the paired shuffled data (p < 10−15, two-way ANOVA, real vs shuffled data, no significant interaction with experimental repeat factor). Each animal’s data are shown as a pale blue line. Overlaid is a mean and SD. Inset, The mean difference in compressibility between each larva’s real and shuffled data. Each larva is shown by a circle, and the orange cross marks the mean. B, The compressibility (y-axis) of the real wild-type data varies non-linearly with uncompressed sequence length. Each larva (of 124) is shown as a dot. C, The number of motifs (y-axis) identified from compressing each wild-type animal’s real and paired shuffled data. Each animal’s data are shown as a pale blue line. Overlaid is a mean and SD. Inset, The mean intra-fish difference in the number of identified motifs. Each larva is shown by a circle, and the orange cross marks the mean. D, Motif length (x-axis) and usage probability (y-axis) across the entire real (blue) and 10 shuffled datasets (black). Note that each shuffled dataset is plotted independently. For each motif length a grey line joins the real and mean shuffled value. E, Each panel shows how Δ compressibility varies in different behavioral contexts. Each pale line shows an individual larva’s average Δ compressibility during the day and the night. The darker overlay shows a population day and night mean and SD. F, Δ Compressibility of 500 module blocks for each wild-type larva, averaged into 1-h time points. Each pale blue line shows 1 of 124 larvae. Line breaks occur when a larva had <500 modules within a given hour. The darker blue overlay shows the mean and SD of these data every hour. Shown are days (white background) and nights (dark background) 5 and 6 of development. Download Figure 3-1, TIF file. [file enu-eN-NWR-0408-19-s06.tif]

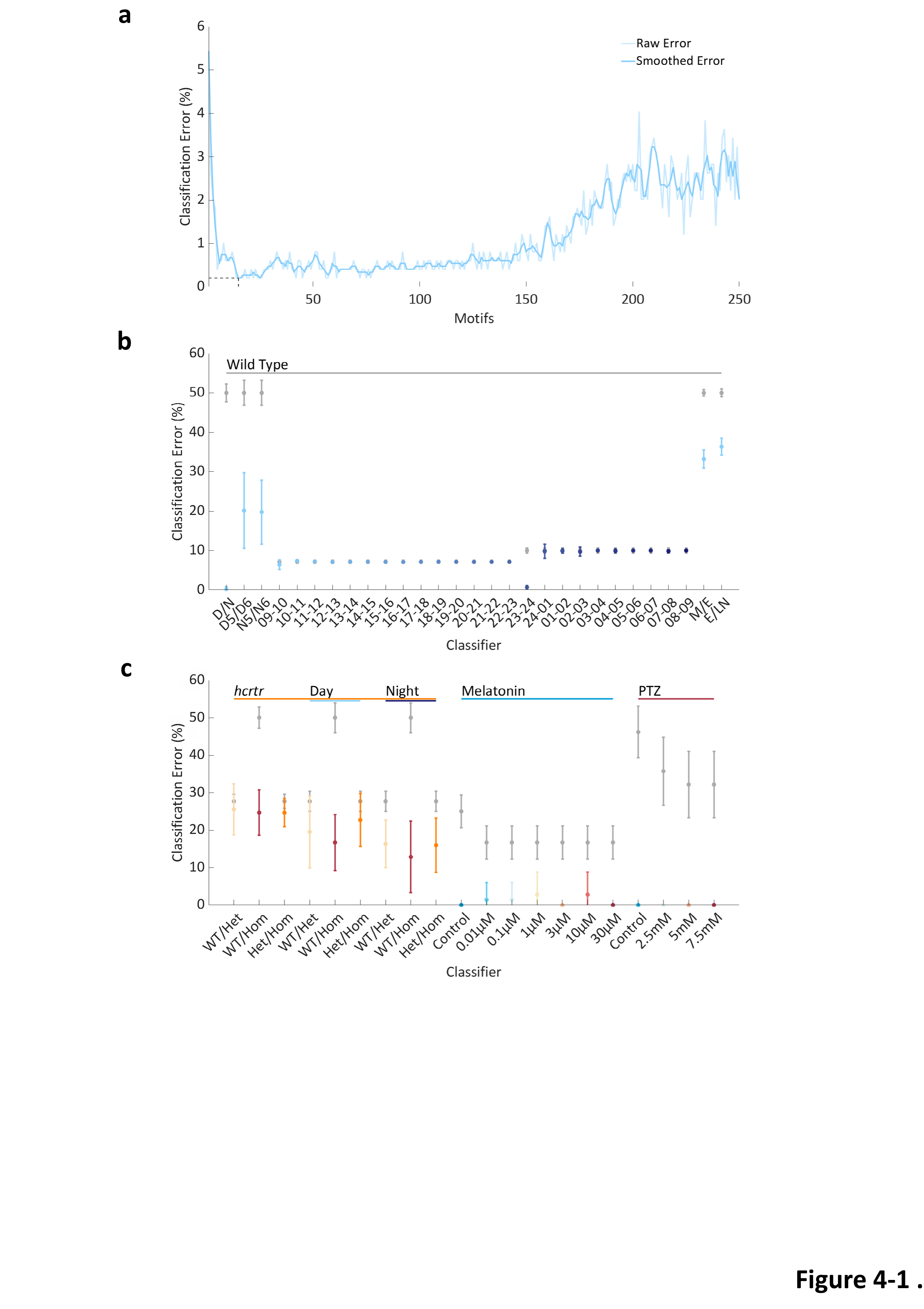

Supplement: Extended Data Figure 4-1 — Motif classifier performance. A, Classification error (%) from linear classifiers separating wild-type day and night behavior using motif enrichment/constraint scores as sequential mRMR motifs from 1 to 250 are added (x-axis). The average error is shown in light blue. Overlaid in darker blue is a running average three motifs wide. The broken black lines show the minimum of the smoothed data to be at 15 motifs, where the classification error is 0.2%. B, Wild-type temporal classifier performance. Real classifiers (color) are shown as a mean and SD from 10-fold cross validation. Majority class classifiers (grey) are shown as value and SE of proportion. Each classifier’s data are listed on the x-axis. D, day; N, night; M/E, morning/evening; E/LN, early/late night. The number of motifs chosen for each classification and exact values for each classifier are detailed in Table 1. C, hcrtr, Melatonin and PTZ classifier performance. Real classifiers (color) are shown as a mean and SD from 10-fold cross validation. Majority class classifiers (grey) are shown as value and SE of proportion. Each classifier’s data are listed on the x-axis. For hcrtr comparisons, grouped classifiers as well as separate day (light blue underline) and night (dark blue underline) classifiers are shown. For melatonin and PTZ, only day data were compared. Classifier details can be found in Table 2. Download Figure 4-1, TIF file. [file enu-eN-NWR-0408-19-s07.tif]
